# Adsorption-Driven Deformation and Footprints of the RBD Proteins in SARS-CoV-2 Variants on Biological and Inanimate Surfaces

**DOI:** 10.1021/acs.jcim.4c00460

**Published:** 2024-07-31

**Authors:** Antonio M. Bosch, Horacio V. Guzman, Rubén Pérez

**Affiliations:** † Departamento de Física Teórica de la Materia Condensada, Universidad Autónoma de Madrid, E-28049 Madrid, Spain; ‡ Condensed Matter Physics Center (IFIMAC), Universidad Autónoma de Madrid, E-28049 Madrid, Spain; § Department of Theoretical Physics, Jožef Stefan Institute, SI-1000 Ljubljana, Slovenia

## Abstract

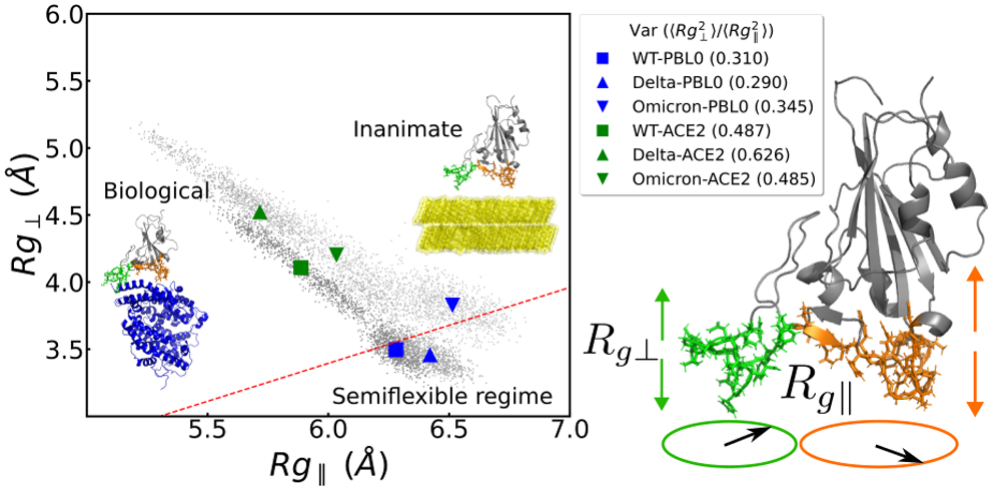

Respiratory viruses, carried through airborne microdroplets, frequently adhere to surfaces, including plastics and metals. However, our understanding of the interactions between viruses and materials remains limited, particularly in scenarios involving polarizable surfaces. Here, we investigate the role of the receptor-binding domain (RBD) of the spike protein mutations on the adsorption of SARS-CoV-2 to hydrophobic and hydrophilic surfaces employing molecular simulations. To contextualize our findings, we contrast the interactions on inanimate surfaces with those on native biological interfaces, specifically the angiotensin-converting enzyme 2. Notably, we identify a 2-fold increase in structural deformations for the protein’s receptor binding motif (RBM) onto inanimate surfaces, indicative of enhanced shock-absorbing mechanisms. Furthermore, the distribution of adsorbed amino acids (landing footprints) on the inanimate surface reveals a distinct regional asymmetry relative to the biological interface, with roughly half of the adsorbed amino acids arranged in opposite sites. In spite of the H-bonds formed at the hydrophilic substrate, the simulations consistently show a higher number of contacts and interfacial area with the hydrophobic surface, where the wild-type RBD adsorbs more strongly than the Delta or Omicron RBDs. In contrast, the adsorption of Delta and Omicron to hydrophilic surfaces was characterized by a distinctive hopping-pattern. The novel shock-absorbing mechanisms identified in the virus adsorption on inanimate surfaces show the embedded high-deformation capacity of the RBD without losing its secondary structure, which could lead to current experimental strategies in the design of virucidal surfaces.

## Introduction

Respiratory viruses are airborne and commonly form microdroplets that can be easily adsorbed onto substrates of different materials, namely, polymers, metals, textiles, and glasses, among other prophylactic materials. The recent pandemic has brought the SARS-CoV-2 virus to the spotlight because of its higher transmission rates.^1−4^ The scientific community has delivered a rapid response in several research fields, from obtaining high-resolution structures of the virus^1,2,5^ to developing vaccines and therapies,^6−8^ passing by several endeavors to elucidate the behavior and weaknesses of the virus via computational virology methods.^9−22^ The transmission modes of the microdroplets^23,24^ enveloping the viruses can be classified into two. Direct transmission takes place when viruses are “caught” airborne, mainly via the nose and mouth, while indirect transmission occurs when they are spread by touching surfaces with functional viruses and moving them into the respiratory system. This second mechanism can be very efficient, as suggested for the wild-type (WT) variant, that they can remain on certain types of surfaces for very prolonged periods of time, up to a few weeks.^4^ Based on this, the World Health Organization (WHO) has presented further recommendations on how to clean surfaces and on the continuous disinfection of hands. Due to the speed of the mutations of SARS-CoV-2, the priority has always been placed on providing insights into the interaction of the receptor-binding domain with angiotensin-converting enzyme 2 (ACE2) for the different mutations. This is a key step in the rapid development of new vaccines or therapies for SARS-CoV-2 mutations.^7^ In this context, the investigation of the interaction of other relevant variants of concern (VoCs) with material surfaces has lagged behind. In fact, little is known about how the Delta- and Omicron variants interact with surfaces.

A computational characterization of the hydrophobic and hydrophilic interactions of the VoCs with different surfaces would provide biophysical insight into current experimental efforts made for developing immobilize (filtering^25^) and also virucide surfaces.^26^

**Figure 1:**
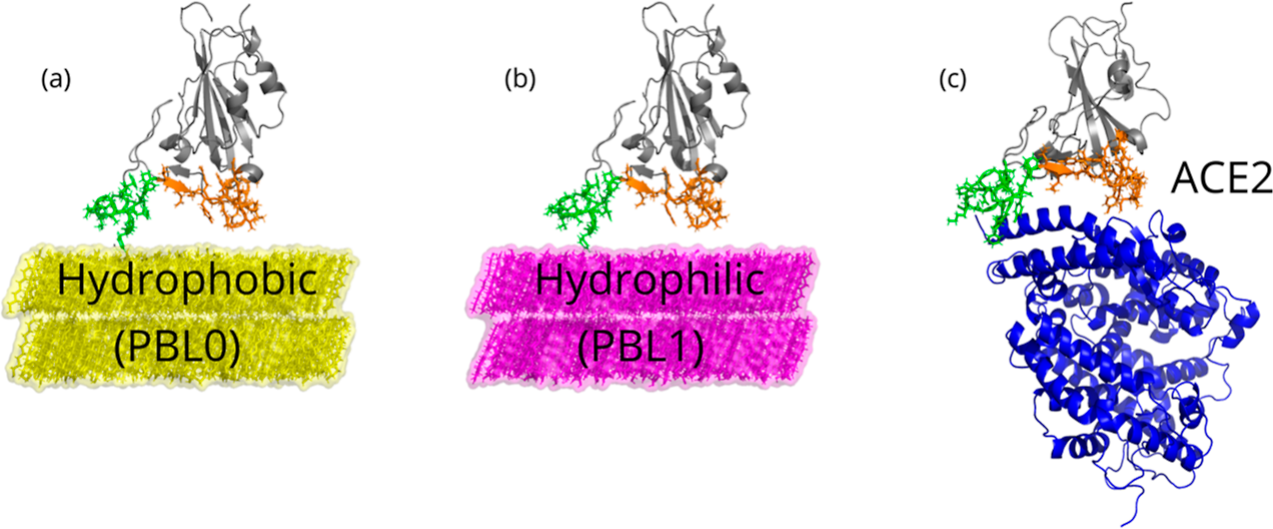
Snapshots of the three main scenarios simulated in this work. (a) Hydrophobic (PBL0) surface with an RBD, (b) hydrophilic (PBL1) surface with an RBD, and (c) RBD-ACE2 complex, as a reference control model, are depicted. Note that in all cases, the left (group 1) and right (group 2) legs of the RBD are colored green and orange, respectively. Water molecules are removed for visualization reasons.

Molecular Dynamics (MD) simulations can provide highly valuable insight into the function of biological systems with high resolution,^16,17,27−29^ and, in particular, on the interaction with different surfaces.^30−69^ From a molecular simulations viewpoint, the comparison of VoCs behavior with different surfaces is still in its infancy. Pioneering studies on relevant hydrophobic, hydrophilic, skin, and coinage surfaces have been performed for the WT spike,^31−33,36^ and recent research has focused only on the RBD interactions to very specific nanomaterials that can be degraded by macrophages.^34^ High-speed AFM (HS-AFM) experiments have demonstrated the enhanced structural flexibility of the RBDs adsorption onto mica substrates.^37^ However, the bottom of the binding domain proteins (in particular, the RBD) is currently arduous to image with HS-AFM techniques. Here, a particular challenge is tracking several binding domain mutations,^38^ like the Omicron variant, which highlights the urge to further elucidate their adsorption mechanisms onto hydrophobic and hydrophilic surfaces by computational biophysics techniques. In this work, we compare three RBDs, namely, those from the WT, Delta, and Omicron variants, interacting with two inanimate surfaces with the same structure and opposite polarities. Our systematic study introduces simplified and polarizable surfaces, modeled in the shape of a molecular bilayer (Polarizable Bilayer or PBL) and characterized by a contact angle that resembles hydrophobic/hydrophilic properties.^39−42^ To contextualize our findings, we contrast the interactions on inanimate surfaces with those on native–biological interfaces, specifically the ACE2 receptor. Including glycans on top of the RBD proteins allowed us to determine their influence on adsorption to inanimate bilayers and explore their differences based on the VoCs. We chose the homogeneous polarizability (less specificity) scheme because it is a stepping stone toward a better understanding of the relationship between the RBD protein hydrophobicity/hydrophilicity mechanisms and their potential binding surfaces. Raising particular interest for surfaces with experimentally measured contact angles, which through nanoscale techniques, e.g., scanning probe microscopy,^43,44^ broadens the application of this research to the codesign of functional materials and complements the electrostatic characterization of the RBDs.^15,21^ The evaluation of adsorption from our simulations covers structural deformation, contact areas, contact histograms, single- and group-based distance analysis, hydrogen bonding (hydrophilic surface), flexibility in 2D (Figure S17), and the formation of possible hydrophobic pockets for the interface sequences of 3 RBDs. The adsorption process of each RBD onto the hydrophobic and hydrophilic surfaces let us group the residues in two regions with a similar amount of residues [see Figure 1, RBD legs in green (group 1) and orange (group 2), and Tables S7 and S8].

## Results

### Morphological Changes during the Adsorption of the RBD onto Polarized Surfaces

**Figure 2:**
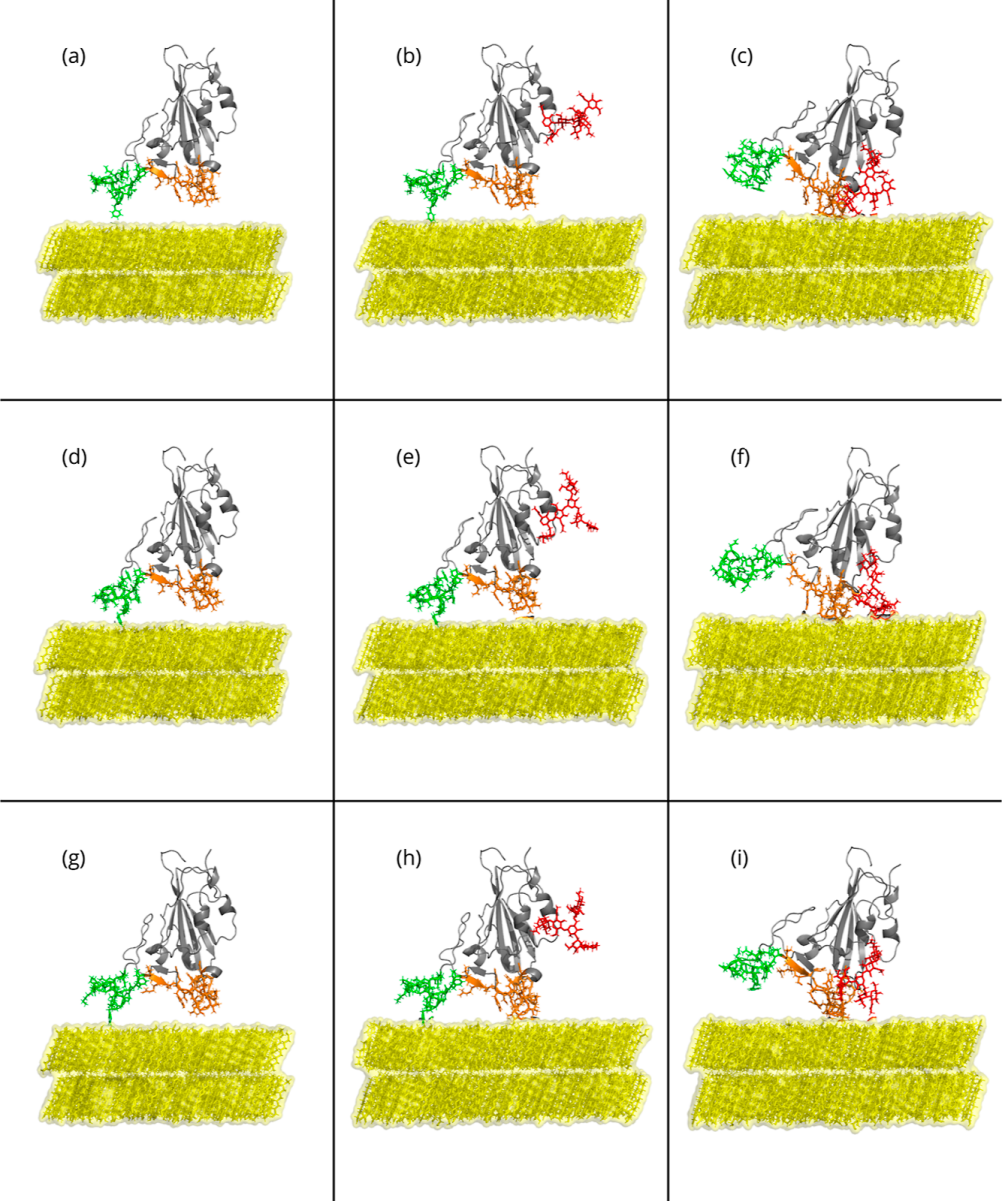
Side-view snapshots of the RBD-PBL simulations performed for this research with the hydrophobic (PBL0) substrate at the beginning of the MD production. Rows show snapshots of the RBDs of (a–c) WT, (d–f) Delta, and (g–i) Omicron with the substrate alone, with its glycan standing vertically to the substrate, and rotated with respect to the substrate with its glycan, from top to bottom. In each panel, the left leg (group 1) is shown in green, the right leg (group 2) is in orange, and PBL0 is shown in yellow. The presence of the glycan chain is depicted in red.

**Figure 3:**
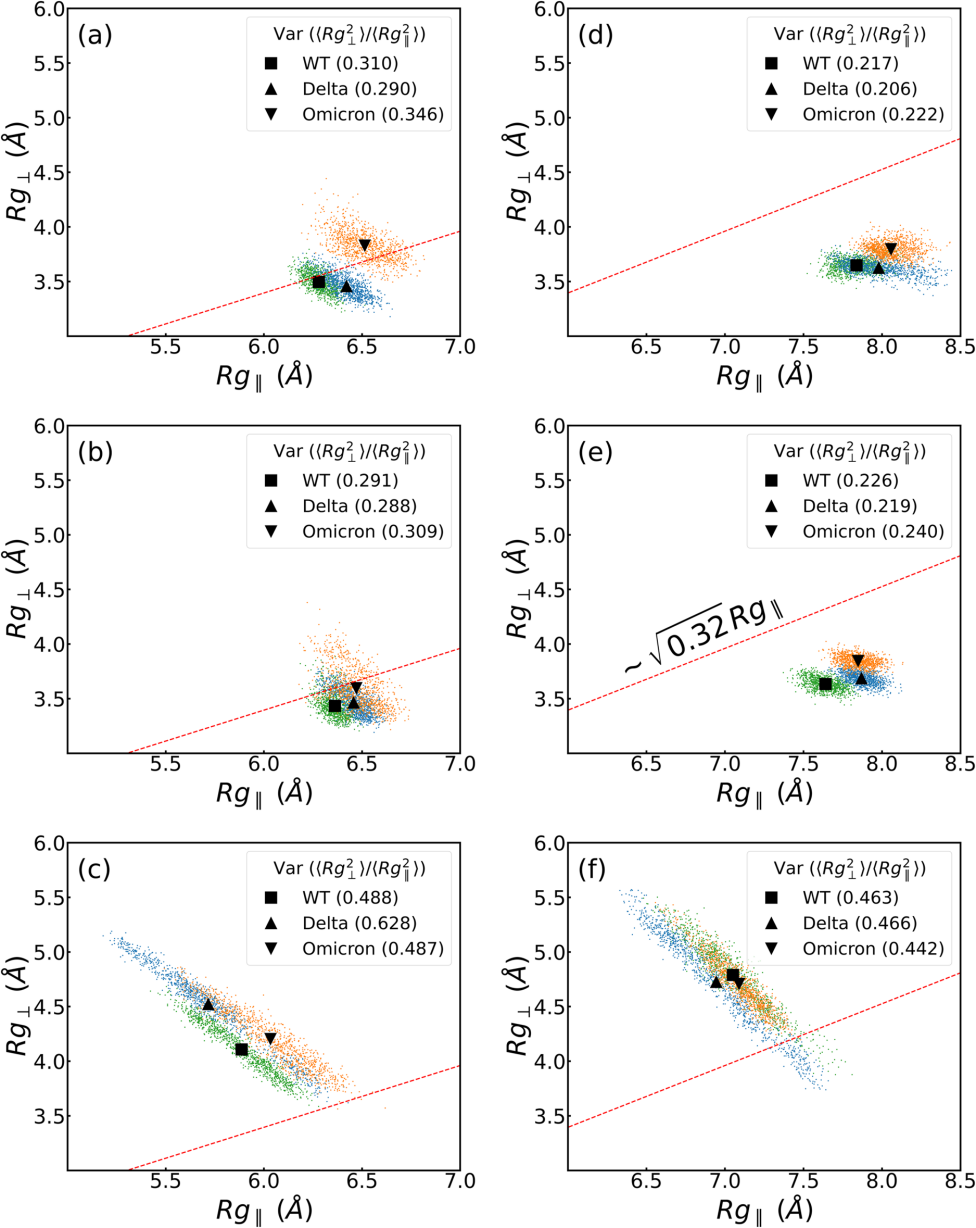
Perpendicular, *R*_g⊥_, vs parallel radius gyration, *R*_g∥_, of (a–c) group 1 and (d–f) group 2 of the RBD in the presence of PBL0, PBL1, and ACE2 (from top to bottom). In each plot, green, blue, and orange dots are values for WT, Delta, and Omicron each 200 ps, respectively, excluding the first 100 ns. The black square, triangle, and inverse triangle are the means of perpendicular and parallel radius gyrations, i.e., ⟨*R*_g⊥_⟩, and ⟨*R*_g∥_⟩ of each variant. In the legends and inside the parentheses, the  ratio is shown for each case.

Figures 2 and S1 show a collection of snapshots of the initial, mid, and final adsorption stages of the RBD interacting with hydrophobic (yellow) and hydrophilic (fuchsia in Figure S1) surfaces, respectively. Three views in each adsorption scenario capture the initial adsorption (1 ns) to the hydrophobic surface, namely, the proteinaceous RBD without glycans (Figure 2a,d,g), RBD with glycans (Figure 2b,e,h), and a rotated-RBD with glycans (Figure 2c,f,i) for the three VoCs. A bottom view of the same adsorption stage of the RBD protein is also shown in Figure S2. For the first and second columns, we clearly observe that both RBD legs (green and orange) are adsorbing onto the surface, with a slight preference for green (group 1). Interestingly, the mid column (with glycans, in vertical RBD) shows a glycan chain (see also the glycan structure in Figure S16) in red that is not directly interacting with the surface as the simulation starts. Hence, in order to further explore the glycan contribution to the adsorption phenomenology of the RBDs, we rotated the molecule into an extreme configuration where the glycan could form more contacts with the modeled surface. The latter shows that glycans are also interacting with the hydrophobic surface, starting from the simulation genesis; however, due to their high flexibility,^45^ they do not remain in contact with the surface. The quantification of morphological changes in the receptor binding motif (RBM) during adsorption is a crucial step to determining the difference in function of the protein due to structural deformation. Figure 3 shows the relationship between the perpendicular and parallel radius of gyration (*R*_g⊥_ and *R*_g∥_, respectively) of the RBM-inanimate (for both groups, as defined in Tables S7 and S8) and RBM-biological (both groups and the ACE2) and allows us a direct comparison among them for each VoC.^46−49^ The interfaces with the PBLs are roughly within the semiflexible polymer regime ().^50,51^ In contrast, the biological interface RBM-ACE2 shows globular behavior. Although both hydrophobic and hydrophilic surfaces are in the same flexibility regime, we observe a difference in *R*_g∥_ between group 1 and group 2, due to the initial conformation of the residues in group 2, which are slightly more elongated (see Figure 3c,f for initial structures between both groups). We highlight that the ratio of the  reaches ca. 3 times by comparing biological to biological–inanimate interfaces, which could trigger irreversible deformations in the RBM as hypothesized elsewhere.^34^

### Interaction between the RBDs and the Polarized Surfaces

**Figure 4:**
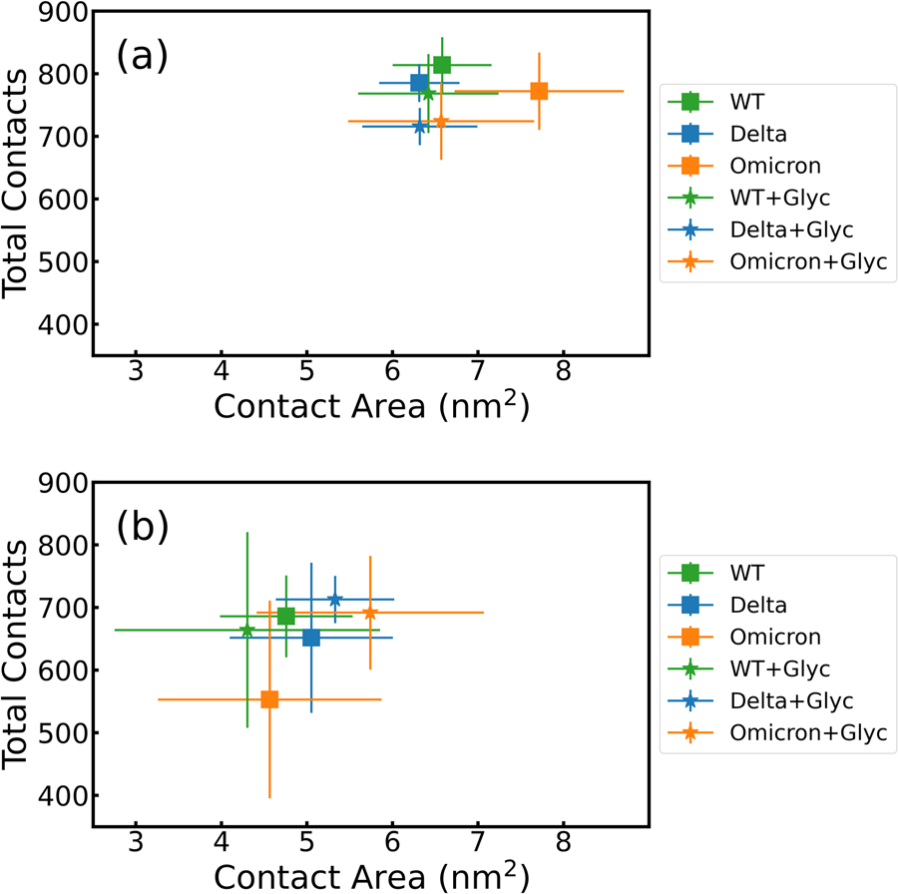
Total contacts per frame vs contact area between the RBDs of WT (green), Delta (blue), and Omicron (orange) and the (a) hydrophobic (PBL0) and (b) hydrophilic (PBL1) surfaces. Squares are the mean over the whole trajectory of RBD-PBLs, and stars are the mean values over the whole trajectory of the RBD-PBLs with glycan simulations. Error bars show the standard deviation over time.

**Figure 5:**
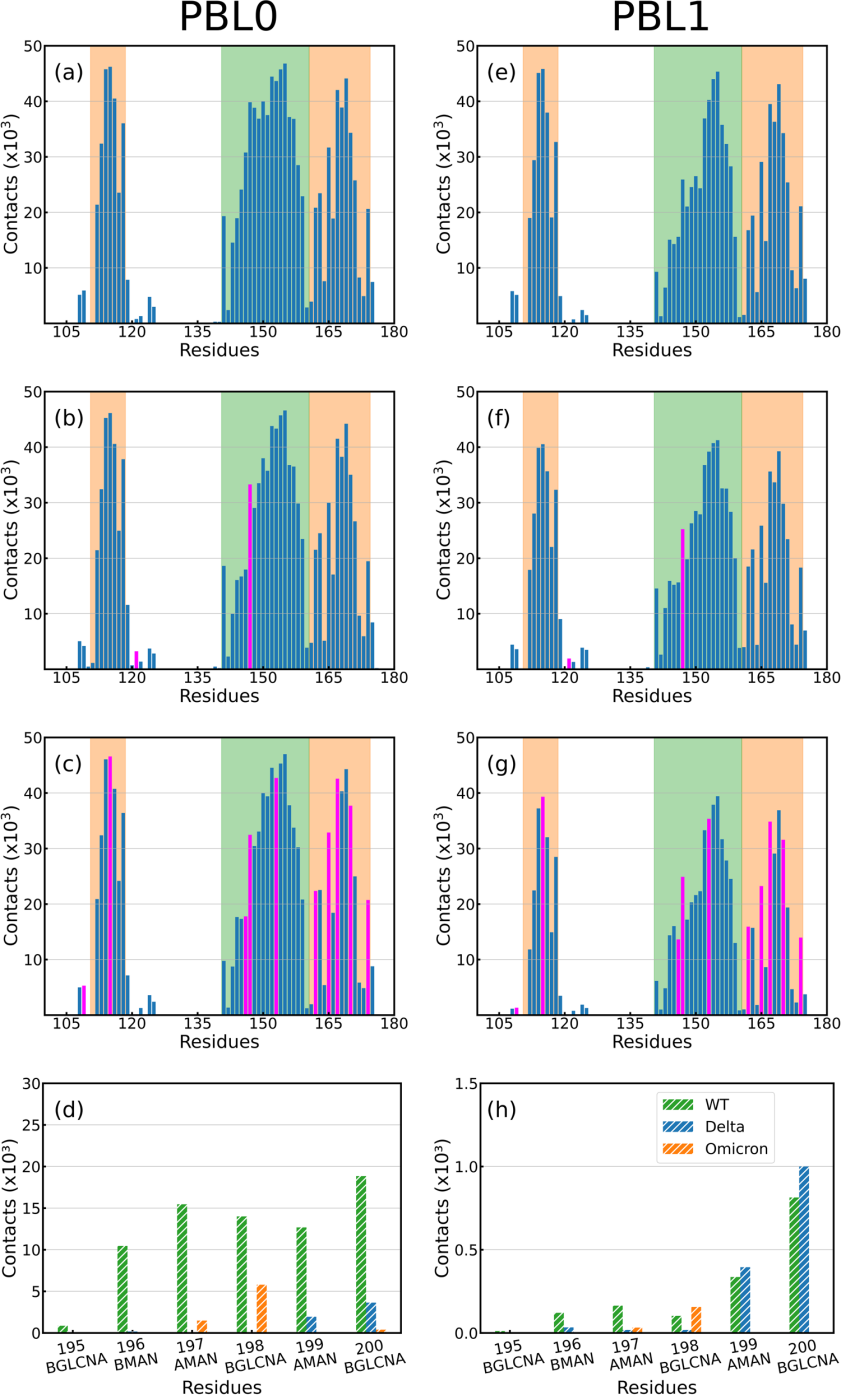
Residue contact histograms between RBDs and (a–d) PBL0 and (e–h) PBL1 for (a,e) WT, (b,f) Delta, and (c,g) Omicron variants. Note that the mutated residues referenced to the WT are colored in fuchsia, and the legs of the RBD are highlighted by color in each region, green (left leg, group 1), and orange (right leg, group 2). (d,h) Contact histogram of the carbohydrates of the glycan to the hydrophobic and hydrophilic surfaces, respectively, in the rotated RBD-PBLs with glycan simulations. Glycan residues 193-BGLCNA and 194-AFUC have been excluded since they do not have contacts with the PBLs.

Two additional physical quantities characterize the adsorption phenomena of the RBDs onto two antipodal surfaces. First, Figure 4 a,b shows the relationship between the number of contacts and the contact area for both surfaces, with and without glycans. In particular, the RBD-PBL0 (Figure 4a) interface has a gain in both the total number of contacts and the contact areas with respect to the RBD-PBL1 interface (Figure 4b). Remarkably, the presence of glycans has a minor effect on both total contacts and contact areas, which lies within the standard deviations (see also Table S1). This can be understood by quantifying the density of glycans in the RBD-up configuration compared to the closed one or further regions of the spike protein, which can be densely populated by glycans,^11^ and hence, they could dominate contact with particular surfaces, like copper ones.^33^ For ACE2, the structures from crystallography provide an initial contact area that is larger than the other two surfaces(see Table S1). Note also that the modeled polarizable surfaces do not contain glycans; those are located only at the RBD. Another observation is the gain in contact area from the hydrophobic surface. This result goes in line with former observations comparing the interaction between the WT spike protein and the graphite and cellulose-modeled surfaces,^31^ also shown in the distance analysis in the distances and contact analysis section and the contact histograms of Figure 5. Most contact areas for the different VoCs are in a similar range, with the exception of the Omicron-PBL0 and Omicron-glycan-PBL1 interface, with a notorious gain of around 1 nm^2^ in each case (Figure 4). Note also that the simulations on PBL1 are also prone to higher standard deviations due to the hopping behavior of the protein on hydrophilic surfaces.

A second quantity is the accumulative number of contacts per residue, Figure 5 a–c,e–g shows the accumulated number of contacts vs RBD residues for the VoCs with both hydrophobic and hydrophilic surfaces. The mutated residues are highlighted in fuchsia, and the RBD regions are also shadowed in green (left region-group 1) and orange (right region-group 2), as previously illustrated in Figure 1. The number of contacts in the hydrophobic interaction (Figure 5a–c) is more frequent than the hydrophilic surface (Figure 5e–g), as reported in the Distances Analysis and Contact Analysis section. The RBD-ACE2 contacts can be found in Figure S3. In addition, Figure 5d,h shows the accumulated number of contacts of the glycan groups onto the PBLs during the simulations of the RBD in rotated conformation (further details can be found in the Supporting Information). We observe a decrease in terms of the number of contacts compared to the protein side of the molecule, especially for the hydrophilic surface. As expected, most contacts between the glycans and the surface are located at the tip of the glycan. However, this interaction may be not only conducted by the enhanced flexibility of glycans but also influenced by the positive charges coming from mutations at the RBD amino acids, in particular for the Omicron variant. Complementary to the latter observation, we have analyzed local charges in the glycans, which may interact with the mutations in group 2 of the Omicron variant, which contains more positive charges^29^ (see Figure S16).

### Distance and Contact Analysis of the RBDs and the Polarized Bilayers

**Figure 6:**
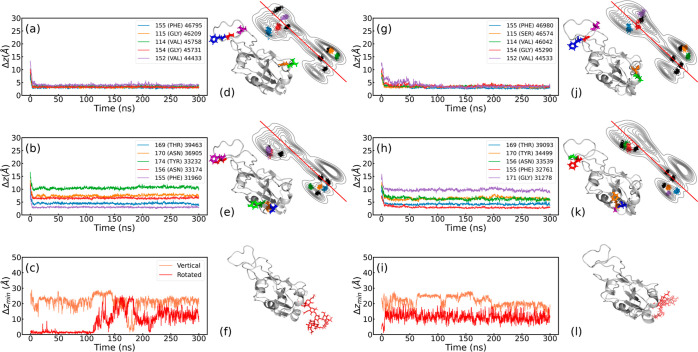
Center of mass distance of the residues of (a,b) WT and (g,h) Omicron RBDs to the hydrophobic substrate of the top 5 residues with most contacts with the (a,g) hydrophobic substrate and (b,h) ACE2. Legends show the residue IDs, the residue names, and the total contacts over the trajectory of each ranked residue (format: ResID (ResName) TotalContacts). The visualization of residue loci for each case is also shown at the bottom of (d,e) for WT-PBL0 and (g,h) for Omicron-PBL0, with colors corresponding to the distance plots (a,b,g,h). The minimum distance between the glycan and the substrate on the hydrophobic surface in the vertical and rotated configurations for (c) WT-PBL0 and (i) Omicron-PBL0. In (f,l), Glycan is shown in red. Note that all snapshots were taken from a bottom perspective. Complementary at the top of (a,b,g,h), we present contour-line plots showing the top 10 residues with most contacts with the (g) hydrophobic substrate and (h) ACE2. Note that in the contour-line plots, the color code of the top 5 corresponds to residue distance plots on their left side, the remaining 5 are depicted in black color.

Figure 6 compiles a detailed analysis of the WT and Omicron, centered in the distances Δ*z* (as defined in the Methods section) of the top 5 residues with most contacts in adsorption with the hydrophobic surface. Note that the cases on the hydrophilic surface and Delta variant are also included in Supporting Information in Figures S4, S7 and S5, S6, respectively. In Figure 6, the first half (left side) refers to the WT variant, while the second half refers to the Omicron, both on hydrophobic PBLs. From left to right, these figures first display the distance curves per residue, next the location of the residues in the RBD protein from a bottom perspective, and the contour-line plots of the RBM, highlighting the center of mass of the top 10 residues, the top 5 in consistent color code with the left side plots, and the next 5 residues in black. The adsorption distance of the WT RBD onto the hydrophobic surface (Figure 6a,b) shows only one coincident residue, PHE-155, out of the total of 5 residues shown in the legend of Figure 6a. The ranks of the legends are given by the contact histograms of PBL0 (Figure 5a) and ACE2 (Figure S3a). The first presents substantial lower distances to the hydrophobic surface. These results are consistent with the contact histograms of Figure 5 and also aim to uncover the differences and coincidences between adsorption between the purely biological and biologically inanimate (biological material) interface, in particular, performing a side-by-side analysis between the RBD-ACE2 and RBD-PBL residues with more contacts. Strikingly, the residue loci of Figure 6d,e illustrate a notorious difference between the two groups of the RBM, where the adsorption (based on the top 5 analysis) in the case of PBL0 is driven by the left region (group 1), while the purely biological interface binding is rather triggered by group 2. Note that the residues with more contacts (based on the histogram of Figure 5) highlight the higher proximity of the residues between the RBD-PBL0 interface and the RBD-ACE2 interface. In addition, next to the WT variant, we provide a direct comparison to the Omicron one. The Omicron variant (Figure 6g,h,j,k) shows a similar number of noncoincident contacts as the WT variant, in total 4 out of 5. However, the mutations play a key role, for instance in the ACE2 surface, switching the tyrosine 174 (WT) by another one (ResIDs 170 in Omicron) much closer to the other two residues, ResIDs: 169, 171 (see Figure 6e). This may suggest a more specific and compact RBM (i.e., contiguous residues with enhance contact, Figure 5c) interaction for the Omicron variant, as also corroborated via extensive studies of the 3 variant binding energies.^15,21^ On the contrary, for the inanimate surface (see Figure 6g), the mutations within the top 5 residues in close contact do not show binding specificity. This alternative approach could bring potential new tools for modeling virucidal-surfaces and interpreting virus sensors, both of which are current applications requiring computational-aided design^52^ of inanimate surfaces. In other words, designing surfaces by grouping different variants (nonbinding-specific), which could be based on a threshold of mutations per RBM group or variant family.^53^ Certainly, our approach could be also used in the other direction for exploiting specificity; however, this will be part of future work. By extending this benchmark to the top 10 (see Figure S10), we found two mutated residues that start interacting with PBL0 (see discussions in the Hydrogen Bonding Evolution section). In addition, we provide contour-line plots of the top 10 residues with most contacts with (j) the hydrophobic substrate and (k) the ACE2. By considering the closest 10 residues (Figures 6j,k and S10a,b) and comparing biological and biological–inanimate interfaces, a trend of opposite (referenced with the solid red line, see Figure 6j,k) contacts in group 2 is observed, which underlines the different landing footprints. Note that the WT variant presents also similar behavior for the landing footprints of the top 10 residues (see Figure 6d,e at the contour lines). On top of this, it helps understand the drastic morphological changes shown by exploring the ratio  differences on both interfaces of Figure 3a,d,c,f. The glycans are another molecule found in the virus RBDs, as expected, these molecules are much more flexible than the protein chains, as shown for all variants onto both surfaces (depicted in the 2D-flexibility maps of Figure S17). In order to naturally induce the adsorption of glycans, we have simulated not only the vertical approach of the RBD but also a rotated case. Interestingly, the rotated WT RBD shows an adsorption period of ca. 100 ns (red curve in Figure 6c) onto the hydrophobic surface, while for the Omicron variant, the Δ*z* distances are constantly above 10 Å (Figure 6i). The 2D-flexibility (Figure S17) and the distance (Figure 6) analysis show that the Omicron variant seems to fluctuate more in the 2D surface’s than the WT, whereby the Omicron glycan remains unadsorbed. Note that the adsorption times of the glycans in contact with the hydrophobic surface for the WT variant are similar to former simulations on graphite surfaces.^31^

**Figure 7:**
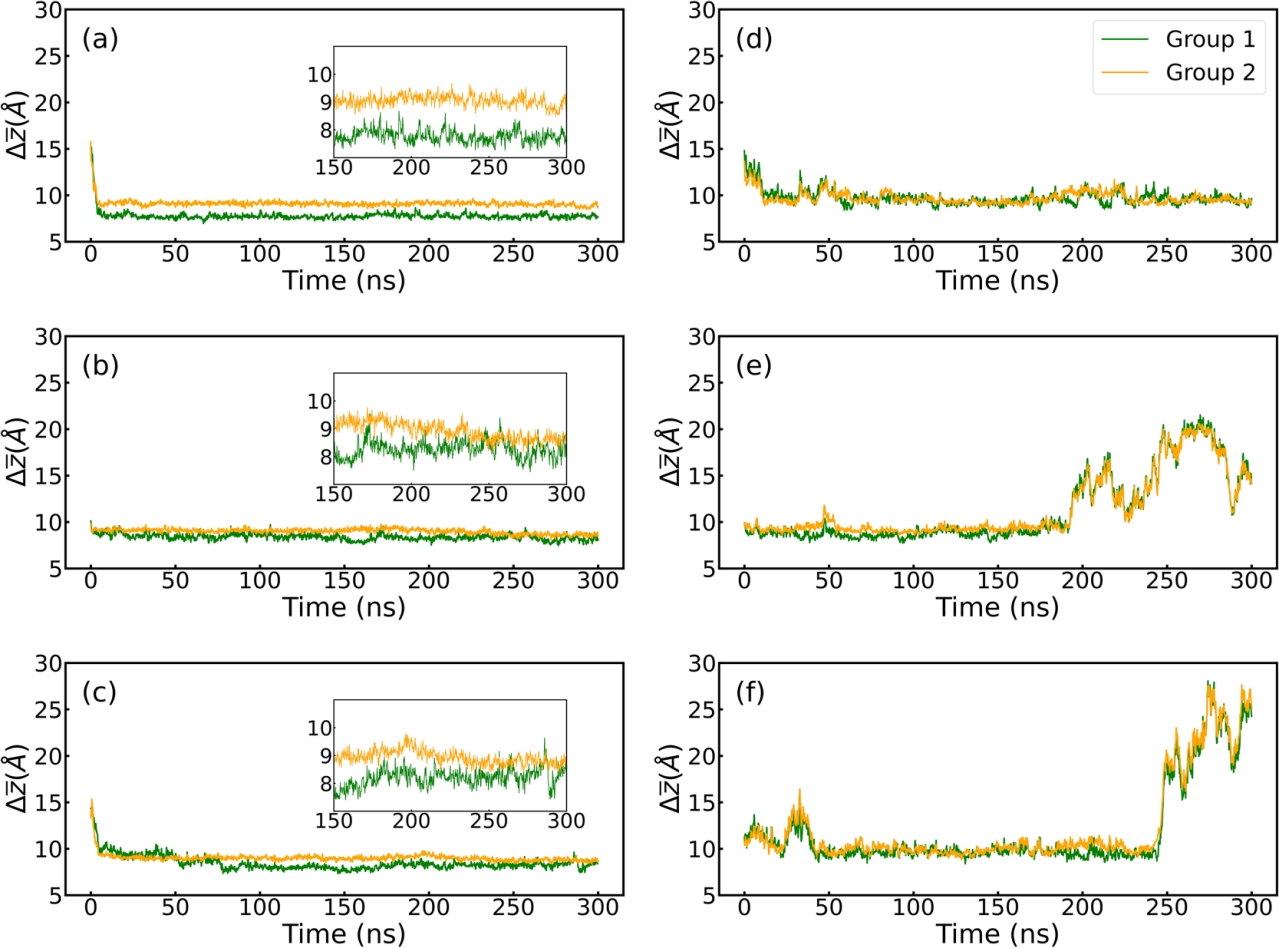
Average distance of the two regions of the RBDs, the left leg in green (group 1) and the right leg in orange (group 2) for the (a–c) hydrophobic and (d–f) hydrophilic surfaces. Variants are ordered as WT, Delta, and Omicron from top to bottom, i.e., (a,d) for WT, (b,e) for Delta, and (c,f) for Omicron. Insets are a zoom of the trajectory from 150 to 300 ns.

Complementary to the top 5 distance analyses, we tackled the distance distribution by both RBD regions defined in green (group 1) and orange (group 2) (Figure 1). For this, we use an average distance from all the residues in each group, which at the same time is the distance of the residue’s center of mass, as defined in the Methods section. Figure 7 shows the distances of the green (group 1) and orange (group 2) regions of the RBD for the WT, Delta, and Omicron variants during their adsorption onto hydrophobic and hydrophilic substrates. Singularly, both regions in the Omicron RBD show similar and sometimes overlapping average distances between both regions (Figure 7c and its inset). This suggests a similar affinity onto the hydrophobic surface for both residue groups, which has been also shown as slightly decreased deformations for the *R*_g⊥_ (see Figure 3). Another important aspect are the mutations in Omicron, whereby group 2 exhibits much more mutations than group 1, which could explain the singular behavior of the Omicron RBD in contrast to its peers (WT and Delta). For WT and Delta on the hydrophobic substrate, group 1 has a slight preference in terms of average distances. Such behavior is, however, adaptable, as shown for the Omicron variant, where the adsorption process starts with slight preference for group 2 and then after ≈80 ns group 1 comes closer to the substrate, which could suggest enhanced flexibility depending on the variant, as recently reported.^28^ The hydrophilic substrates show a different adsorption pattern in terms of both increased average distances and hopping behavior while landing (Figure 7e,f). Supporting Information on the average values of the simulation trajectories shown in Figure 7 can be found in Table S2. Another complementary analysis to the top 5 residues includes the detailed next 5 residues, which builds up an analysis of the top 10 adsorbed residues in Figures S8–S13. This analysis improves the understanding of the described hopping or balanced landing behavior during adsorption onto hydrophobic and hydrophilic substrates beyond average distance values.

**Figure 8:**
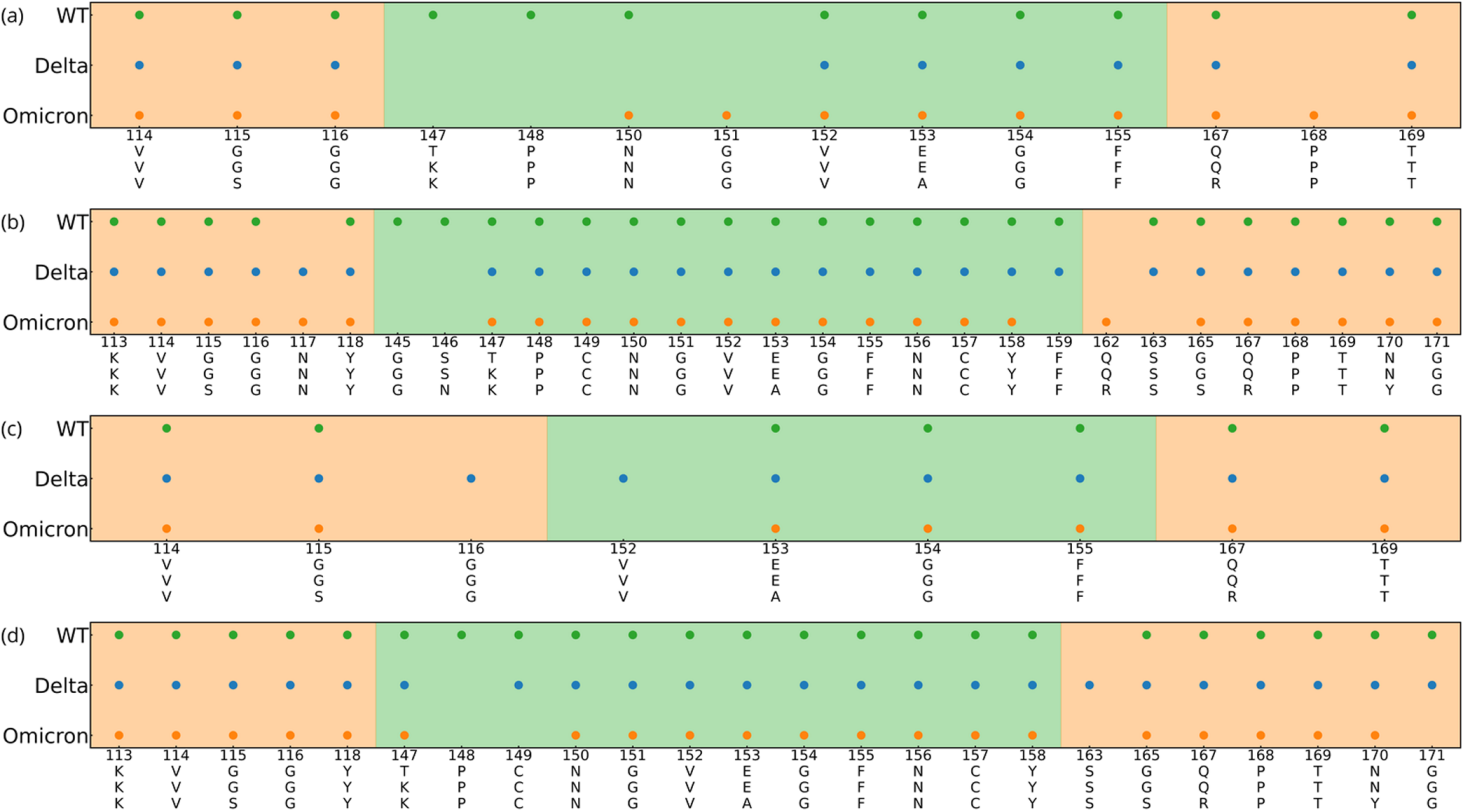
Residues with an average center of mass-surface distance smaller than or equal to 6 Å in (a,c) and 10 Å in (b,d). (a,b) correspond to simulations with a hydrophobic surface and (c,d) correspond to a hydrophilic surface. Note that all the distances plotted here are average distances of the total trajectory, excluding the first 100 ns, and distance values larger than 15 Å.

Summarizing the distance analysis, we show in Figure 8 that residue adsorption at the interface is defined by distance thresholds of 6 Å (Figure 8a,c) and 10 Å (Figure 8b,d). As a general observation, we found more residues at the hydrophobic interfaces than at the hydrophilic ones. Moreover, the differences between variants for the hydrophobic surface are always around 7% (of the total number of residues), which shows a general similar adsorption process. However, this percentage changes once we look at the RBD legs (groups of residues), where Omicron has 1 more residue placed closer to the surfaces than the WT or Delta for group 2 (Figure 8a) and an opposite behavior of Omicron with fewer residues (1 less residue with respect to WT) at such a distance from this surface for group 1 (Figure 8a). At the 10 Å distance threshold (Figure 8b), Omicron’s group 2 levels off with Delta and has only a minimal difference with WT. The landscape differs in group 1, where Omicron reaches ≈23% less residues than the WT and only 2 residues difference with Delta. Switching polarities to the hydrophilic substrate, we found a gain in residues for the WT variant over the other Omicron but only for the 10 Å threshold. In particular, the Delta variant, shows a ≈30% more residues found at the 6 Å threshold. While the second threshold, 10 Å equalizes the difference between WT and Delta. Now comparing the numbers between hydrophobic and hydrophilic surfaces for WT and Omicron at the closest threshold of 6 Å, we can determine also the ratio of 2 with more close contact residues at the hydrophobic interface. This might suggest a more reactive behavior on hydrophilic of group 1 of the RBM, which impede the collective adsorption of the WT and Omicron RBDs the RBD to completely polar surfaces. A similar analysis has been previously performed.^54^ An interconnected analysis of Figure 8c,d spots the hydrogen bonds formed at the hydrophilic surface (see the Hydrogen Bonding Evolution and Hydrophobic Formations at the Interfaces RBD with Polarized Surfaces section) and their distribution in the different RBD footprint.

To finalize this section, we analyzed the ratios between the residues found at different interfaces to the surface (Table 1), showing the ratio between the number of residues at the interface distances (6, 10, and 14 Å) averaged out from the last 280 ns of the trajectory (*N*_avg_) (see Figure 8), and *N*_min_ is the number of residues found at the interface at any time of the same trajectory, which fulfills the same distance criteria. The interfaces are defined by 3 threshold distances to the surface: 6, 10, and 14 Å. Table 1 shows the ratio *N*_min_/*N*_avg_ at those 3 threshold distances, at the hydrophobic surface (left side) and hydrophilic surface (right side). In most cases, the following rule applies: the further the distance threshold, the closer the values between *N*_min_ and *N*_avg_, and, hence, their ratio tends to unity. Connecting the 3 threshold distances provides a sort of dynamic insight into the adsorption process, which can serve as input for the estimation of other distance-dependent types of interactions in max and average ranges, such as electrostatic forces^18^ and van der Waals.^55−57^

**Table 1:** Ratios of the Number of Residues with Minimum Distance and the Average Distance, *N*_min_/*N*_avg_, for Smaller or Equal to Average Distances 6, 10, and 14 Å to PBL0 (Hydrophobic) and PBL1 (Hydrophilic)

	PBL0			PBL1		
ratio (*N*_min_/*N*_avg_)	dis. 6 Å	dis. 10 Å	dis. 14 Å	dis. 6 Å	dis. 10 Å	dis. 14 Å
WT	1.83	1.37	1.19	2.57	1.43	1.17
Delta	2.56	1.54	1.18	2.11	1.57	1.15
Omicron	1.92	1.56	1.21	2.86	1.80	1.31

At the same time, this analysis reinforces our previous description of a generalized hopping adsorption of the RBD molecule onto hydrophilic surfaces by observing that the overall sporadic closeness to the surface is much more frequent for PBL1. Hence, the values in Table 1 are generally higher for the hydrophilic surface. Remarkably, by using these ratios, we can also discern at which distance the adsorption to the surface tends to reach a plateau. For instance, it can be noticed that for PBL0, plateaus are about to be reached for WT and Delta.

Connected to Table 1, we present Tables S5 and S6, which show the *N*_min_/*N*_avg_ normalized by the maximum number of contacts, and Tables S3 and S4 show those normalization factors.

### Hydrogen Bonding Evolution and Hydrophobic Formations at the RBD Interfaces with Polarized Surfaces

**Figure 9:**
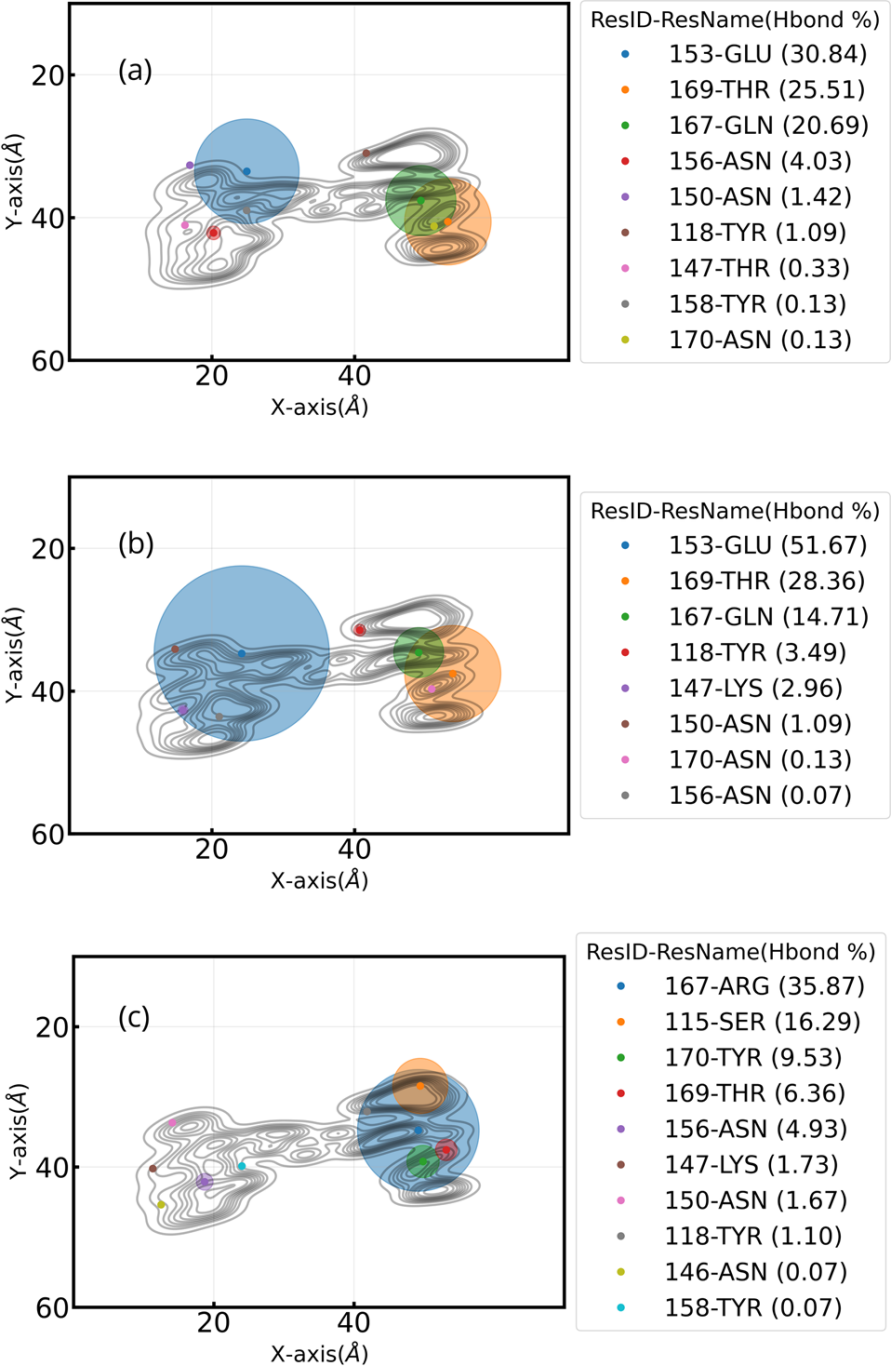
H-bonds of residues with hydrophilic surfaces for (a) WT, (b) Delta, and (c) Omicron. A scatter plot shows the location of all centers of mass of hydrophilic residues that have H-bonds with the surface from a top view. Circles represent the percentage of the trajectory in which each residue has H-bonds. Scatter colors vary according to the ranking of this percentage. In the legend, in parentheses next to the ResIDs and residue names, the percentage of time with H-bonds is shown for each residue. Note that all plots here include the contour-line plots.

In this section, we show first the hydrogen bonds formed at the interface between the three VoCs and the hydrophilic surface as averages over time. Figure 9 shows all residues that form H-bonds per variant of concern. Here, we rapidly identify the location of the H-bonds by including the contour lines of the different RBDs. Strikingly, the mutations from hydrophobic to hydrophilic residues in the Omicron variant are directly spotted in group 2 (right side of the plot), reaching ≈70% of the total H-bonds which is consistent with the literature.^58^ In terms of hydrogen bonds, the WT variant is almost perfectly balanced by 50% in both RBM regions (left and right). The ResIDs found in the top 10 distance analysis are also found in the H-bond criteria. Similar analyses for the WT variant have also reported the presence of H-bonds with hydrophilic surfaces.^31^

**Figure 10:**
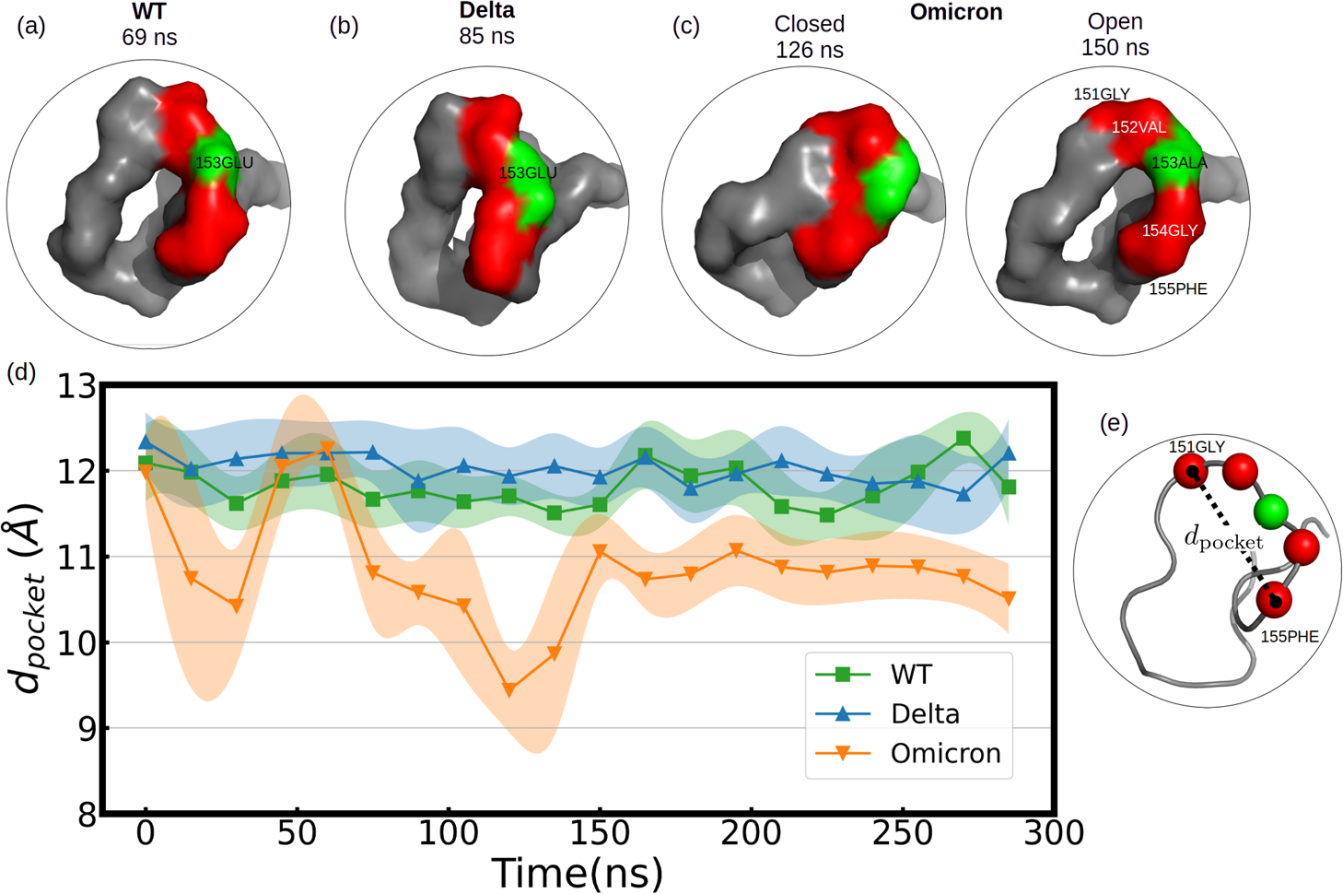
(a–c) Crook-handle formation in group 1 in the presence of a hydrophobic surface. In red, the loci of the C-alpha atoms corresponding to the residues 151, 152, 154, and 155 are represented; in green is residue 153, which mutates from WT to Omicron. Residue 153 is part of the crook-handle, forming a hydrophobic pocket in Omicron. (d) Mean C-alpha atom distance between residues 151-GLY and 155-PHE over each 15 ns, *d*_pocket_, which is depicted in (e). Colored shadows represent the standard error over these 15 ns. Note that the perimeter of the crook-handle from residue 151-GLY to 155-PHE was 15.3 ± 0.1 Å for all variants. In Omicron, a breathing mechanism is promoted [see in (c) that at 126 ns closed state and open at 150 ns] due to the attraction of 153-ALA to the hydrophobic surface. The adsorption of 153-ALA into PBL0 forces 154-GLY to be reoriented to the 149-CYS direction for geometrical reasons.

We analyzed the formation of ring-like structures and, in perspective of the distance from the surface, rather crook-handle-like structures. In Figure 10d, we show those formations for the different variants and the hydrophobic surface represented by a collective variable *d*_pocket_ (see Figure 10e) as the simulation time evolves. After noticing that the RBM’s group 1 is the most flexible region, we analyzed the formation in time of crook-handle-like structures for the different variants (Figure 10) with the hydrophobic surface defining and using a collective variable, *d*_pocket_ (Figure 10d and Table S9). Remarkably, residue 153 mutates from hydrophilic (in WT-GLU) to hydrophobic (in Omicron ALA) and allows the formation of hydrophobic pockets as the crook-handle forms its closed configuration (see Figure 10c). This hydrophobic formation could potentially drive further design of rather nanopatterned surfaces to trap and disassemble viruses, similar to the ideas discussed for killing cells with nanostructured surfaces.^59,60^

## Discussion

We have presented extensive MD simulations of three SARS-CoV-2 receptor binding domains with two different model surfaces. These simplified surfaces have been implemented in order to distinguish the adsorption processes of the WT, Delta, and Omicron RBDs, driven by homogeneous hydrophobic and hydrophilic surfaces. In addition, as a reference to determine the “biological contacts” of the RBD, we simulated the RBD-ACE2 interfaces of the tackled variants. Initially, the RBDs adsorb to both surfaces through residues, which are mostly located in the RBM region. From this point, the adsorption on each surface describes substantially different patterns. Overall, the RBDs adsorption onto hydrophobic surfaces shows a gain in the number of residues at the interface, contact area, and contact histograms compared to the hydrophilic one. However, in detail, each RBD variant presents complex and different surface-adsorption mechanisms. Considering two regions of adsorption at the RBM surface interface with the same total amount of residues, we show how the regions balance out (hops) during adsorption and favor contact formation. Specifically, the WT-RBD and hydrophobic surfaces present enhanced adsorption over the other two peers, quantified in terms of the average distances of the RBM surface and contact histograms. While for the hydrophilic surface, WT is also presenting better adsorption, as quantified by both the average distances of the RBM surface and the location of the H-bonds distributed along the whole RBM contact region. In contrast, the adsorption onto hydrophilic surfaces for Delta and Omicron shows intermittent contact, where the H-bonds are mostly distributed on one of the ends of the RBM. The average group distances show for both Delta and Omicron a distinguishable oscillatory behavior onto hydrophilic surfaces, especially for Omicron, where the H-bonds are 90% localized in the RBM’s group 2, resulting in a hinged-oscillatory mechanism, consistent with recent structural biology insights in the literature.^61^

The simulations were also used to compare the adsorption trends of the residues in contact between RBDs-ACE2 (biological contacts) and RBDs-PBLs (inanimate/material interfaces). Such an analysis provides important differences between the preferred adsorption regions of the RBM, depending on the biological (specific) and the inanimate (nonspecific, purely hydrophobic or hydrophilic surfaces). Our results show systematically that the RBM’s group 2 (right leg) adsorption takes place in residues (roughly half of the adsorbed amino acids) of opposite domains depending on the biological or nonbiological origin of the surfaces. In the RBM’s group 1, there are also changes in the adsorption footprints between inanimate and biological surfaces; however, they are not as systematic as in group 2. In fact, those differences in landing-footprints can also explain the RBDs enhanced adsorption-driven deformation, which exhibits deformation ratios in the 2-fold order (/ 2), where the flat hydrophobic and hydrophilic surfaces result in more deformations of the protein.

We have presented different landing footprints and deformation mechanisms of the mutations in the RBDs proteins that can be directly applied for the interpretation of experiments; for example, the recent HS-AFM work performed for RBDs to ACE-2.^37^ Future assessments for the design of virucidal surfaces supported by novel HS-AFM techniques are a perfect match for future collaborations. It is evident that our study has to be treated as a first step in understanding the molecular adsorption between VoCs and surfaces. Nonetheless, it sheds light on the complex adsorption-driven deformation of the proteins by considering hydrophobic and hydrophilic interactions, which are key to understanding short-range binding. Strikingly, the RBM is capable of deforming up to two times more at a surface with only minor changes in their secondary structure (see Figure S15), suggesting a shock-absorbing mechanism.

Another aspect to discuss is the interaction of glycans with inanimate surfaces. While our results show that the contacts are mostly driven by the amino acids located in the RBM, the glycans included in the RBD initial configurations are also forming contacts with the modeled surfaces (see Figure 5d,h). In terms of the interaction times, they are similar to those found for the spike glycoprotein on a graphite surface.^31^ Nonetheless, in a different simulation setup,^33^ namely, the spike protein model interacting with metal surfaces, it has been shown that the collective motion of glycans covering the surface of the spike protein could be the leading interaction overcoming the one with amino acids. Such observations highlight the importance of studying modeled surfaces, which characterize the short-range interactions (hydrophilic/hydrophobic) with surfaces characterized by measurable quantities such as contact angles. In this way, the interface between fully solvated proteins and inanimate surfaces can be studied and also compared to further experiments, such as HS-AFM. In future work, our method to calculate contact angles of specific surfaces can be employed for precise surface characterization.^41^

Beyond the scope of this article, our results can be combined with current multiscale models of the whole virus^19,20^ to further study the dynamics of the spike protein landing process and the shock-absorbing features we observed for the RBMs. Molecular simulation of whole viruses would also provide insight into the amount of spike proteins per surface area, considering the flexibility and membrane translocation properties of the SARS-CoV-2 envelope.

## Methods

### Molecular Dynamics

The simulations in this research do not consider the whole spike for the VoCs because it has been shown in the WT that most interactions with surfaces were identified at the RBD,^31^ more specifically in the RBM, which is the region of the spike protein interfaced to ACE2.^13^ We explicitly include the RBD in the simulations and mimic the interaction of the RBD with the rest of the spike by introducing some mechanical constraints at the contacts between the RBD and the S1 region, as explained later in this section.

All-atom simulations were carried out with Gromacs 2023,^62^ and the system components (protein, ions, and the polarizable bilayer) were modeled using the CHARMM36^63,64^ force field and TIP3P^65^ for the water. CHARMM-GUI was used to join the glycan to the RBDs. Energy minimization used CPUs, while all production runs used 1× GPU, as the former scaled better than 2× GPUs for our systems. All RBD models for the initial configurations were adopted from previously published results by Barroso and co-workers,^15^ where we added the disulfide bonds to the VoCs. The hydrophobic (PBL0) and hydrophilic (PBL1) surfaces were built from a small patch of decanol (DOL), in which restraints were used in order to maintain the bilayer shape and avoid any effects of the mechanical properties of the surface. As discussed in previous works,^39−41^ the bilayer model does not include any curvature (is not flexible) and also no molecular defects. The replicated PBL (using *gmx editconf*) patch was solvated in a slab-formed water box. The RBD models for WT, Delta, and Omicron were added to an 8 nm × 8 nm × 12 nm cubic box containing the bilayers. The polarity of the OH- groups of the DOL chains was tuned to 0 or 1 for hydrophobic and hydrophilic surfaces, respectively.^41^ This procedure was repeated 3 times for each polarity, aggregating from 3 replicas per VoC (WT, Delta, and Omicron), obtaining 18 configurations. The same procedure was followed for the RBDs, with ACE2 also using cubic boxes. All simulations included ions that worked under neutral charge conditions. Periodic boundary conditions were applied, and PME was used for long-range electrostatics. Minimization was done by the steepest descent (50,000 steps) with an integration step of 0.01 ps. The equilibration time for the *NVT* and *NPT* was 100 ps, respectively. For each RBD, we determined the contacts between the RBD and the S1 region. Those contacts were applied as position restraints of 250 kJ mol^–1^ nm^–2^ in the *x* and *y* axes. In other words, we considered as flexible regions of the RBD all the others that are not in contact with S1. However, in order to quantify adsorption, we kept the *z*-axis free of restraints in all of the RBD-S1 contacts. Note also that the position restraints do not apply to the RBM interface (see also Figure S14 and Table S10). Production simulations began from the final equilibrated snapshots, and three copies (with angular rotations of up to 3° from the reference) of each system were simulated. Finally, 300 ns trajectories of each replica were collected, as described in Table 2. Note that all production runs used an integration step of 2 fs. Starting configurations from the MD production can be found in a Zenodo repository.^66^

**Table 2:** General Configurations of the Simulated Systems^a^

VoC	no. of replicas	PBL polarities	box size (nm^3^)	atoms	time per replica [ns]
WT	3	2	7.5 × 7.5 × 12	2980	300
Delta	3	2	7.5 × 7.5 × 12	2993	300
Omicron	3	2	7.5 × 7.5 × 12	3036	300
WT +Glyc	2 (1r + 1v)	2	8.5 × 7.6 × 12	2979 + 192	300
Delta +Glyc	2 (1r + 1v)	2	8.5 × 7.6 × 12	2992 + 192	300
Omicron +Glyc	2 (1r + 1v)	2	8.5 × 7.6 × 12	3035 + 192	300
WT + ACE2	3		15.2 × 15.2 × 15.2	12,510	300
Delta + ACE2	3		15.2 × 15.2 × 15.2	12,523	300
Omicron + ACE2	3		15.2 × 15.2 × 15.2	12,564	300

a Note that 576 decanol molecules were used in systems without glycans, and 648 decanol molecules in simulations with glycans. Simulations of RBD-PBLs with glycans are done in two different configurations of the RBD with respect to the PBL; a vertical RBD (1v) and a rotated RBD (1r) configuration. Snapshots of the initial configurations for PBL0 are shown in Figure 2.

### Structural Analysis

In all cases, the radius of gyration parallel (*R*_g∥_) and perpendicular (*R*_g⊥_) were calculated using the equations detailed in the Supporting Information.

Moreover, the contact area has been calculated by subtracting (SASA_RBD_ + SASA_PBL_ – SASA_RBD+PBL_)/2 and (SASA_RBD_ + SASA_ACE2_ – SASA_RBD+ACE2_)/2 for the PBL and ACE2 simulations, respectively.

### Contact Analysis

Two types of contact analysis have been considered, one using the no. of contacts vs simulation time and the other no. of contacts vs residues. The latter includes the highest-resolution trajectory available and sums up the contacts found with the PBL. While the no. of contacts vs simulation time includes temporal averages of the contacts every 1 ns, this facilitates the comparison with the distance analysis presented in the next paragraph. We have computed the accumulative contacts per residue over the whole trajectory for the histograms and summed them to the total contacts of the RBD with the PBLs for the total contacts in (Figure 4). Contacts were counted at each frame when the distance of the center of mass of residues to the surfaces (both PBLs or the ACE2) was less than 14 Å.

### Distance Analysis

Given the simplified definition of the polarizable bilayer, we provide an in-house distance analysis that shows the explicit distance of the center of mass to the PBL, according to
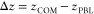
1where *z*_COM_ is the position of the center of mass of each residue and *z*_PBL_ is the position of the bilayer. This quantity is calculated every 200 ps, once per snapshot.

## Data Availability

All-atom simulations were carried out with Gromacs 2023; corresponding parameter files, input files, topologies, position restraints, and initial configurations, as well as the analysis scripts as Jupyter notebooks and the data produced in this work, are available on the Zenodo repository. https://zenodo.org/records/12760554.

## Supporting Information Available

RBD-PBL snapshots for the hydrophilic substrate; bottom view snapshots of the RBDs adsorbed onto both substrates; mean values with STD of the contact area calculations; contact histograms of the RBD-ACE2 interactions; top 5 COM distances for the Delta variant onto both hydrophilic and hydrophobic surfaces; top 5 COM distances for the 3 variants onto both hydrophilic and hydrophobic surfaces; residues involved in groups 1 and 2 for the analysis; illustration of the position restraints applied to all RBDs and the corresponding table; and plot containing the distributions of secondary structures for the RBD alone and RBDs onto modeled surfaces (PDF)
