# Supplementary material for: Adsorption-Driven Deformation and Footprints of the RBD Proteins in SARS-CoV-2 Variants on Biological and Inanimate Surfaces
Source: J Chem Inf Model. 2024 Jul 31;64(15):5977–90. doi: 10.1021/acs.jcim.4c00460 (PMC11323246; doi:10.1021/acs.jcim.4c00460)
Supplement: Supplementary file 1 — ci4c00460_si_001.pdf [file ci4c00460_si_001.pdf]

Supplementary Material: Adsorption-driven deformation and footprints of the RBD proteins in SARS-CoV-2 variants on biological and inanimate surfaces

Antonio Bosch[o,†] Horacio V. Guzman[o,*,†,¶] and Rubén Pérez[*,†]

†*Departamento de Física Teórica de la Materia Condensada, Universidad Autónoma de Madrid, E-28049 Madrid, Spain*

‡*Condensed Matter Physics Center (IFIMAC), Universidad Autónoma de Madrid, E-28049 Madrid, Spain*

¶*Department of Theoretical Physics, Jožef Stefan Institute, SI-1000 Ljubljana, Slovenia*

[o] *These authors share first authorship.*

E-mail: horacio.guzman@uam.es; ruben.perez@uam.es

Contact Analysis

In Figure 5 the accumulative contacts of each residue have been computed with the following 5 steps:

1 Center of mass(COM) of the residues are computed

2 The interface of surface of the PBLs is considered to be represented only by the oxygen atoms of the hydroxyl (OH^-) group of decanol. Therefore, the is only one atom of each decanol residue as reference of the surface.

3 Having the positions of the COM of each residue and the positions of the oxygen atoms in the decanol, the distance between each COM residue (reference coordinate) and each decanol Oxygen atom (configuration coordinate) is computed using `distances.distance_array` function from the MDAnalysis package (Click for function documentation).

4 At each frame, if any of these distances is smaller than 14 Å, is counted as a contact. Therefore, one RBM residue can have more than one contact in each frame.

5 Finally, for the histograms, the contacts over the whole trajectory is summed for each residue.

Structural Analysis

In all cases, we have calculated the Radius of Gyration parallel ($R_{g\|}$) and perpendicular ($R_{g\perp}$) were calculated as follows,

$$R_{g\|} = \sqrt{\frac{1}{N}\sum_i m_i \left[(x_i - x_{CM})^2 + (y_i - y_{CM})^2\right]} \quad (1)$$

$$R_{g\perp} = \sqrt{\frac{1}{N}\sum_i m_i (z_i - z_{CM})^2}, \quad (2)$$

where $\mathbf{R}_{CM} = (x_{CM},\, y_{CM},\, z_{CM})$ is the position of the center of mass, m_i the mass of each residue and N the total number of residues.

Supplementary Tables and Figures

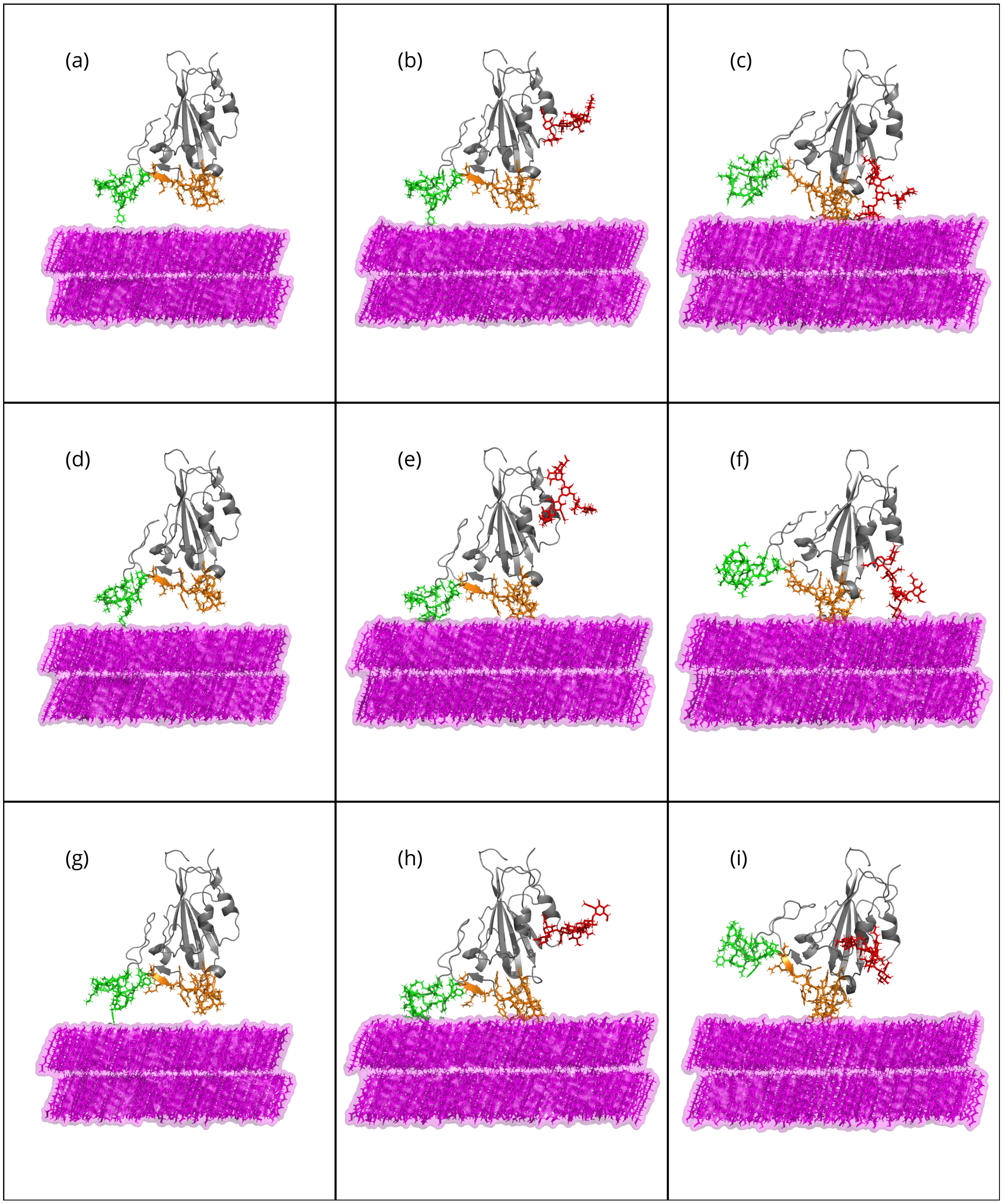

Figure S1: Side view snapshots of the RBD-PBL simulations performed for this research with the hydrophilic (PBL1) substrate at the beginning of the MD production. Rows show snapshots of the RBDs of (a-c) WT,(d-f) Delta, and (g-i) Omicron with the substrate alone, standing vertically to the substrate with its glycan, and rotated respect to the substrate with glycan.

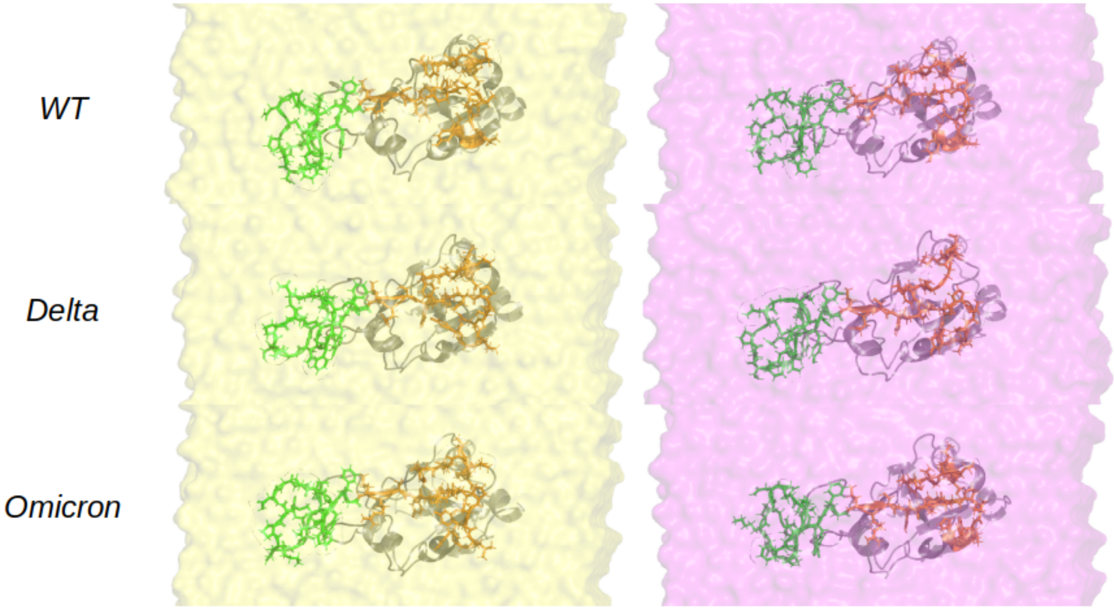

Figure S2: Bottom snapshots of the adsorption of RBDs to the hydrophobic (left) and hydrophilic (right) after 300ns. Ordered as WT, delta, omicron from top to bottom.

Table S1: Table showing the mean contact area, in nm^2, over the last 200ns (and their corresponding standard deviation) of the WT, Delta and Omicron-RBDs in presence of the PBL0, PBL1 and ACE2. Data of the simulations in presence of PBL0 and PBL1 with Glycans attached in the RBDs are also added.

Mean ± Std (nm^2)	RBD-PBL0	RBD-PBL1	RBD-PBL0 (vGlyc)	RBD-PBL1 (vGlyc)	RBD-ACE2
WT	7 ± 1	5 ± 1	6 ± 1	4 ± 2	9 ± 1
Delta	6.3 ± 0.5	5 ± 1	6 ± 1	5 ± 1	9 ± 1
Omicron	8 ± 1	5 ± 1	7 ± 1	6 ± 1	9.8 ± 0.4

Table S2: Table showing the mean distance (and standard deviation), in Å, of the group regions 1 and 2 during the adsorption over the last 200 ns for the WT, Delta, and Omicron RBDs in presence of PBL0, and PBL1. Adsorption to the surface are considered only in distances lower than 15 Å.

	Group 1	Group 2
WT-PBL0	7.7 ± 0.2	9.1 ± 0.2
Delta-PBL0	8.3 ± 0.3	8.9 ± 0.3
Omicron-PBL0	8.1 ± 0.3	8.9 ± 0.2
WT-PBL1	9.5 ± 0.5	9.6 ± 0.5
Delta-PBL1	10 ± 2	11 ± 2
Omicron-PBL1	9.7 ± 0.6	10.0 ± 0.6

Table S3: Table contains Normalization factor $N_{\mathrm{var}}/N_{\mathrm{maxvar}}$ for distances 6 Å, 10 Å, 14 Å from PBL0, where N_{var} and N_{maxvar} are the number of residues for each variant within the distance ranges and the number of residues within the distance ranges of the variant with maximum residues.

	Dist. 6 Å	Dist. 10 Å	Dist. 14 Å
WT	1.00	1.00	0.96
Delta	0.75	0.96	1.00
Omicron	1.00	0.93	0.96

Table S4: Table contains Normalization factor $N_{\mathrm{var}}/N_{\mathrm{maxvar}}$ for distances 6 Å, 10 Å, 14 Å from PBL1, where N_{var} and N_{maxvar} are the number of residues for each variant within the distance ranges and the number of residues within the distance ranges of the variant with maximum residues.

	Dist. 6 Å	Dist. 10 Å	Dist. 14 Å
WT	1.00	1.00	1.00
Delta	0.57	0.82	1.00
Omicron	0.71	0.91	0.90

Table S5: Table contains Normalization factor $N_{\mathrm{var}}/N_{\mathrm{maxvar}}$ for distances 6 Å, 10 Å, 14 Å from PBL0, where N_{var} and N_{maxvar} are the number of residues for each variant within the distance ranges and the number of residues within the distance ranges of the variant with maximum residues.

	Dist. 6 Å	Dist. 10 Å	Dist. 14 Å
WT	1.00	1.00	0.96
Delta	0.75	0.96	1.00
Omicron	1.00	0.93	0.96

Table S6: Table contains Normalization factor $N_{\mathrm{var}}/N_{\mathrm{maxvar}}$ for distances 6 Å, 10 Å, 14 Å from PBL1, where N_{var} and N_{maxvar} are the number of residues for each variant within the distance ranges and the number of residues within the distance ranges of the variant with maximum residues.

	Dist. 6 Å	Dist. 10 Å	Dist. 14 Å
WT	0.78	1.00	0.98
Delta	1.00	1.00	1.00
Omicron	0.78	0.87	0.88

Table S7: Table contains normalized ratio values for PBL0, using normalization factors on Table S5.

	Dist. 6 Å	Dist. 10 Å	Dist. 14 Å
WT	1.83	1.37	1.14
Delta	1.92	1.48	1.18
Omicron	1.92	1.44	1.16

Table S8: Table contains normalized ratio values for PBL1, using normalization factors on Table S6.

	Dist. 6 Å	Dist. 10 Å	Dist. 14 Å
WT	2.00	1.43	1.15
Delta	2.11	1.57	1.15
Omicron	2.22	1.57	1.15

The Glycan Reader sequence (GRS) of the used Glycans is:

1 BGLCNA

2 - 16A:AFUC

3 - 14B:BGLCNA

4 - - 14B:BMAN

5 - - - 16A:AMAN

6 - - - -12B:BGLCNA

7 - - - 13A:AMAN

8 - - - - 12B:BGLCNA

Table S9: Table contains the ResIDs and corresponding residue names of residues used in Group 1. Mutations are highlighted in fuchsia. **CN**: Charged Negative, **CP**: Charged Positive, **UP**: Uncharged Polar and **NP**: NonPolar

ResIDs	WT resnames	Delta resnames	Omicron resnames
141	ILE (NP)	ILE (NP)	ILE (NP)
142	TYR (UP)	TYR (UP)	TYR (UP)
143	GLN (UP)	GLN (UP)	GLN (UP)
144	ALA (NP)	ALA (NP)	ALA (NP)
145	GLY (NP)	GLY (NP)	GLY (NP)
146	SER (UP)	SER (UP)	**ASN (UP)**
147	THR (UP)	**LYS (CP)**	**LYS (CP)**
148	PRO (NP)	PRO (NP)	PRO (NP)
149	CYS (NP)	CYS (NP)	CYS (NP)
150	ASN (UP)	ASN (UP)	ASN (UP)
151	GLY (NP)	GLY (NP)	GLY (NP)
152	VAL (NP)	VAL (NP)	VAL (NP)
153	GLU (CN)	GLU (CN)	**ALA (NP)**
154	GLY (NP)	GLY (NP)	GLY (NP)
155	PHE (NP)	PHE (NP)	PHE (NP)
156	ASN (UP)	ASN (UP)	ASN (UP)
157	CYS (NP)	CYS (NP)	CYS (NP)
158	TYR (UP)	TYR (UP)	TYR (UP)
159	PHE (NP)	PHE (NP)	PHE (NP)
160	PRO (NP)	PRO (NP)	PRO (NP)

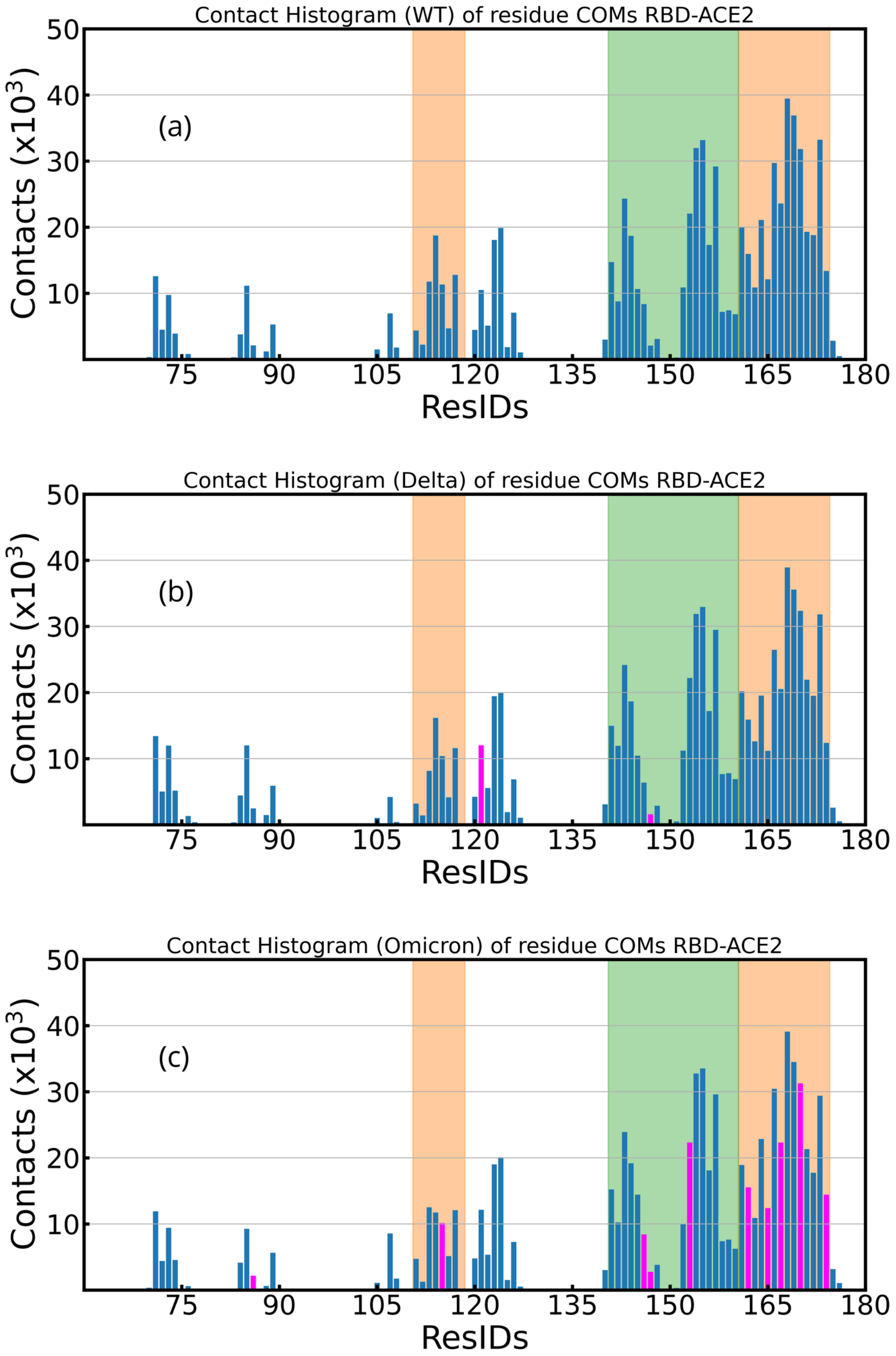

Figure S3: The histograms of contacts between the (a) WT, (b) delta, and (o) omicron RBDs and the ACE2. Note that the residue mutations to the WT variant are colored in fuchsia.

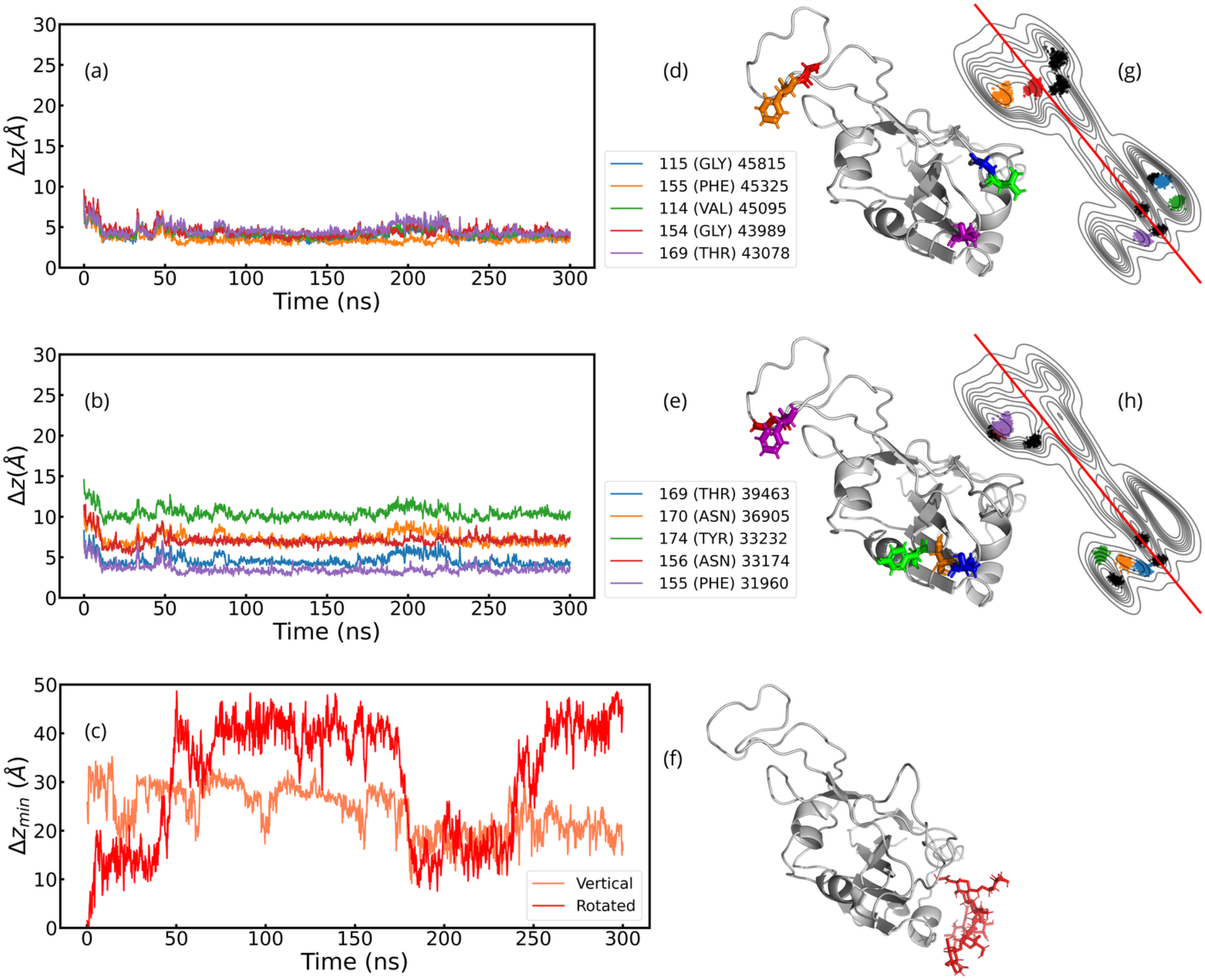

Figure S4: (a,b) Center of mass distance of the residues of WT-RBD to the hydrophilic substrate of the top 5 residues with most contacts with (a) the hydrophilic substrate and (b) the ACE2. Legends show the ResIDs, the residue names and the total contacts over the trajectory of each ranked residue (format: ResID (ResName) TotalContacts). Visualization of residues loci are also shown in (d) and (e) with colors corresponding with the distance plots (a-b). (c) shows the minimum distance between the glycan and the substrate in the hydrophilic surface in a vertical and rotated configuration. In (f), the glycan is shown in red. Note that all snapshots in this plot were taken from a bottom perspective. Complementary to (d) and (e), we present KDE contour-line plots of top 10 residues with most contacts with (g) the hydrophobic substrate and (h) the ACE2. Note that in plots (g) and (h) the color code of the top 5 correspond to plots (d) and (e), the remaining 5 residues have adopted black color.

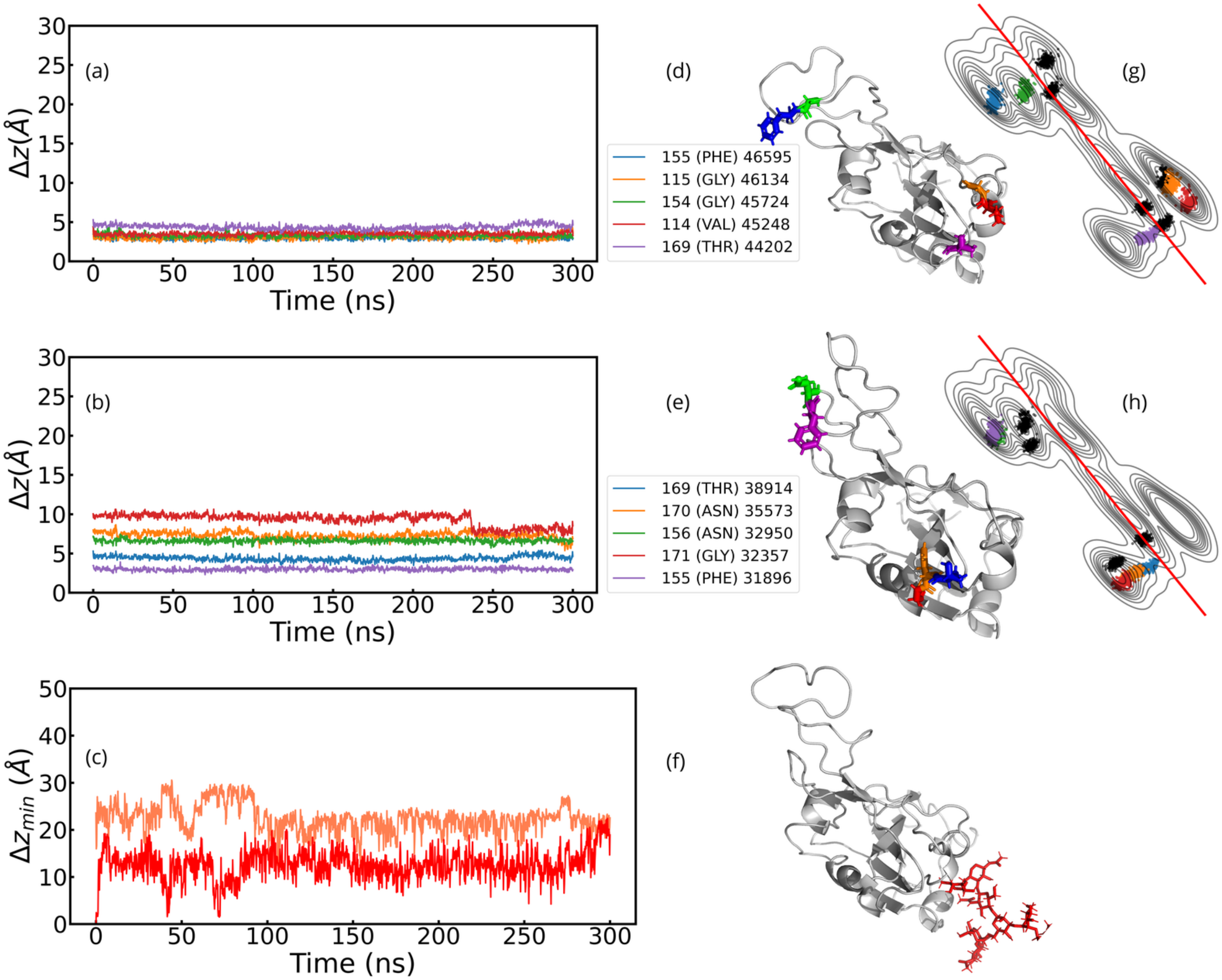

Figure S5: (a,b) Center of mass distance of the residues of Delta-RBD to the hydrophobic substrate of the top 5 residues with most contacts with (a) the hydrophobic substrate and (b) the ACE2. Legends show the ResIDs, the residue names and the total contacts over the trajectory of each ranked residue (format: ResID-ResName TotalContacts). Visualization of residues loci are also shown in (d) and (e) with colors corresponding with the distance plots (a-b). (c) shows the minimum distance between the glycan and the substrate in the hydrophobic surface in a vertical and rotated configuration. In (f), the glycan is shown in red. Note that all snapshots in this plot were taken from a bottom perspective. Complementary to (d) and (e), we present KDE contour-line plots of top 10 residues with most contacts with (g) the hydrophobic substrate and (h) the ACE2. Note that in plots (g) and (h) the color code of the top 5 correspond to plots (d) and (e), the remaining 5 residues have adopted black color.

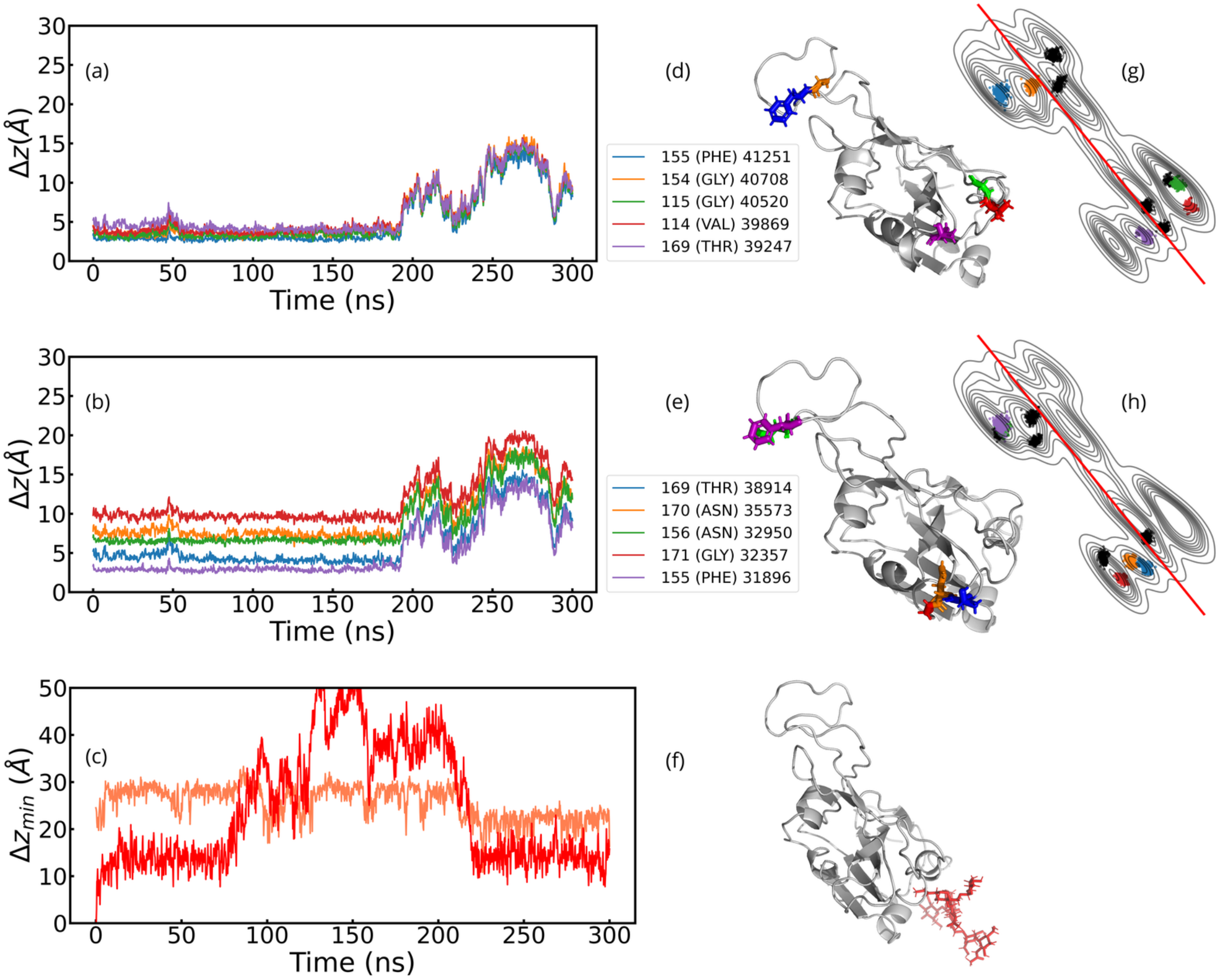

Figure S6: (a,b) Center of mass distance of the residues of Delta-RBD to the hydrophilic substrate of the top 5 residues with most contacts with (a) the hydrophilic substrate and (b) the ACE2. Legends show the ResIDs, the residue names and the total contacts over the trajectory of each ranked residue (format: ResID-ResName TotalContacts). Visualization of residues loci are also shown in (d) and (e) with colors corresponding with the distance plots (a-b). (c) shows the minimum distance between the glycan and the substrate in the hydrophilic surface in a vertical and rotated configuration. In (f), the glycan is shown in red. Note that all snapshots in this plot were taken from a bottom perspective. Complementary to (d) and (e), we present KDE contour-line plots of top 10 residues with most contacts with (g) the hydrophobic substrate and (h) the ACE2. Note that in plots (g) and (h) the color code of the top 5 correspond to plots (d) and (e), the remaining 5 residues have adopted black color.

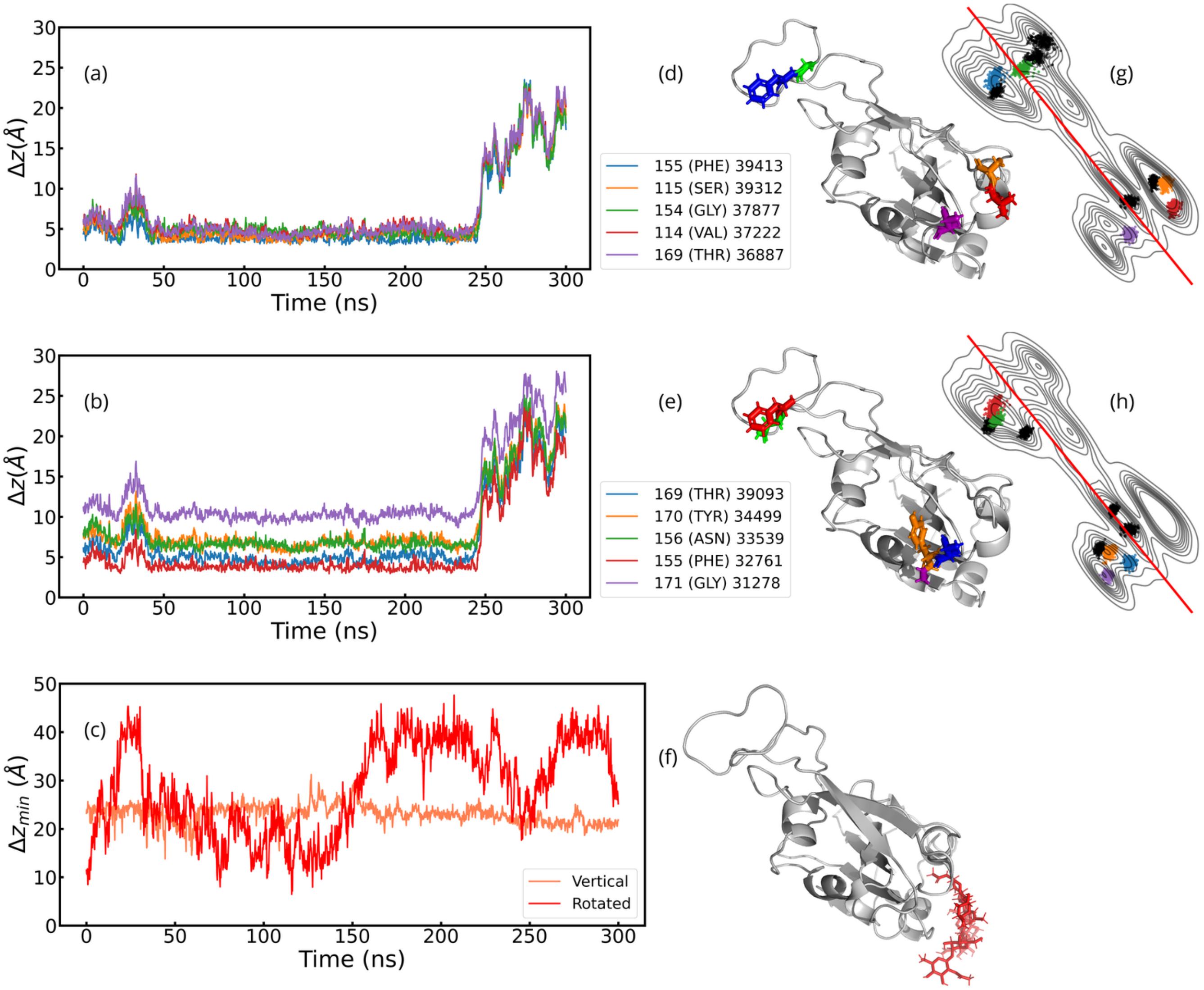

Figure S7: (a,b) Center of mass distance of the residues of Omicron-RBD to the hydrophilic substrate of the top 5 residues with most contacts with (a) the hydrophilic substrate and (b) the ACE2. Legends show the ResIDs, the residue names and the total contacts over the trajectory of each ranked residue (format: ResID (ResName) Total Contacts). Visualization of residues loci are also shown in (d) and (e) with colors corresponding with the distance plots (a-b). (c) shows the minimum distance between the glycan and the substrate in the hydrophilic surface in a vertical and rotated configuration. In (f), the glycan is shown in red. Note that all snapshots in this plot were taken from a bottom perspective. Complementary to (d) and (e), we present contour-line plots of top 10 residues with most contacts with (g) the hydrophobic substrate and (h) the ACE2. Note that in plots (g) and (h) the color code of the top 5 correspond to plots (d) and (e), the remaining 5 residues have adopted black color.

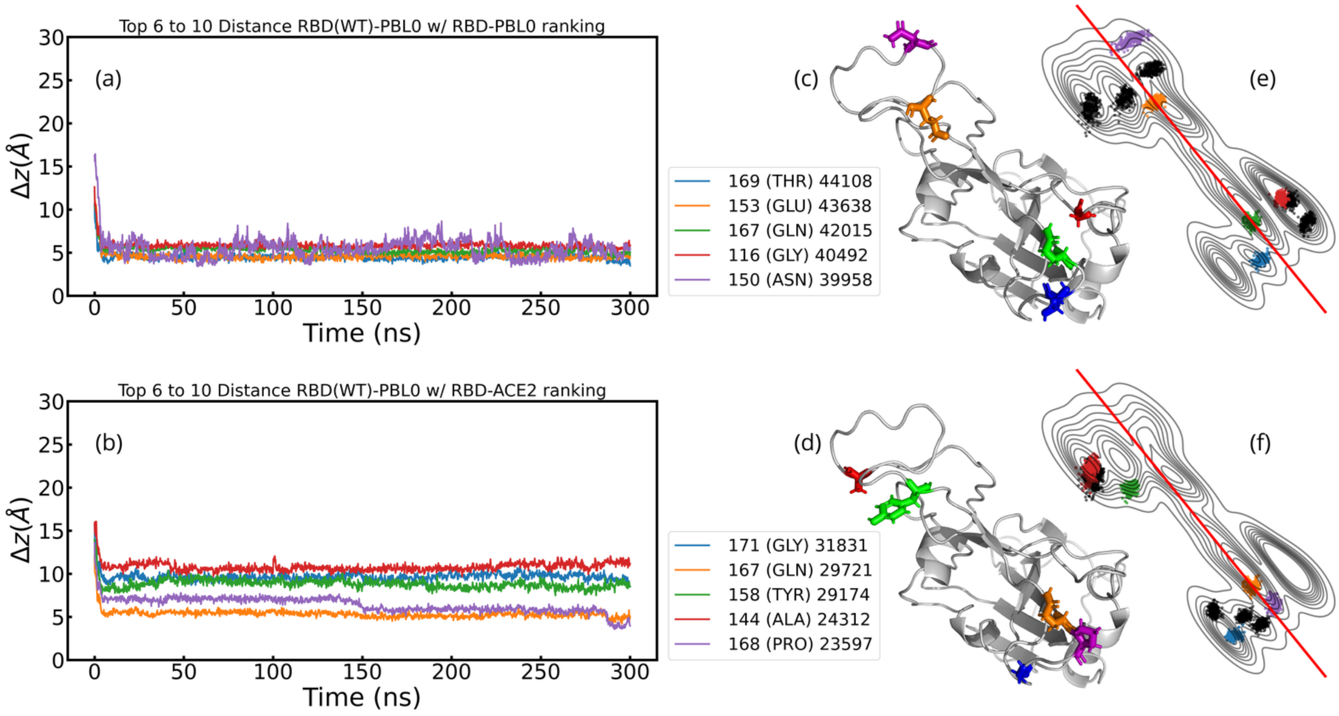

Figure S8: (a,b) Center of mass distance of the residues of WT-RBD to the hydrophobic substrate of the top 6 to 10 residues with most contacts with (a) the hydrophobic substrate and (b) the ACE2. Legends show the ResIDs, the residue names and the total contacts over the trajectory of each ranked residue (format: ResID-ResName TotalContacts). Visualization of residues loci are also shown in (c) and (d) with colors corresponding with the distance plots (a-b). Complementary to (d) and (e), we present KDE contour-line plots of top 10 residues with most contacts with (g) the hydrophobic substrate and (h) the ACE2. Note that in plots (g) and (h) the color code of the top 6 to 10 residues correspond to plots (d) and (e), the top 5 residues have adopted black color.

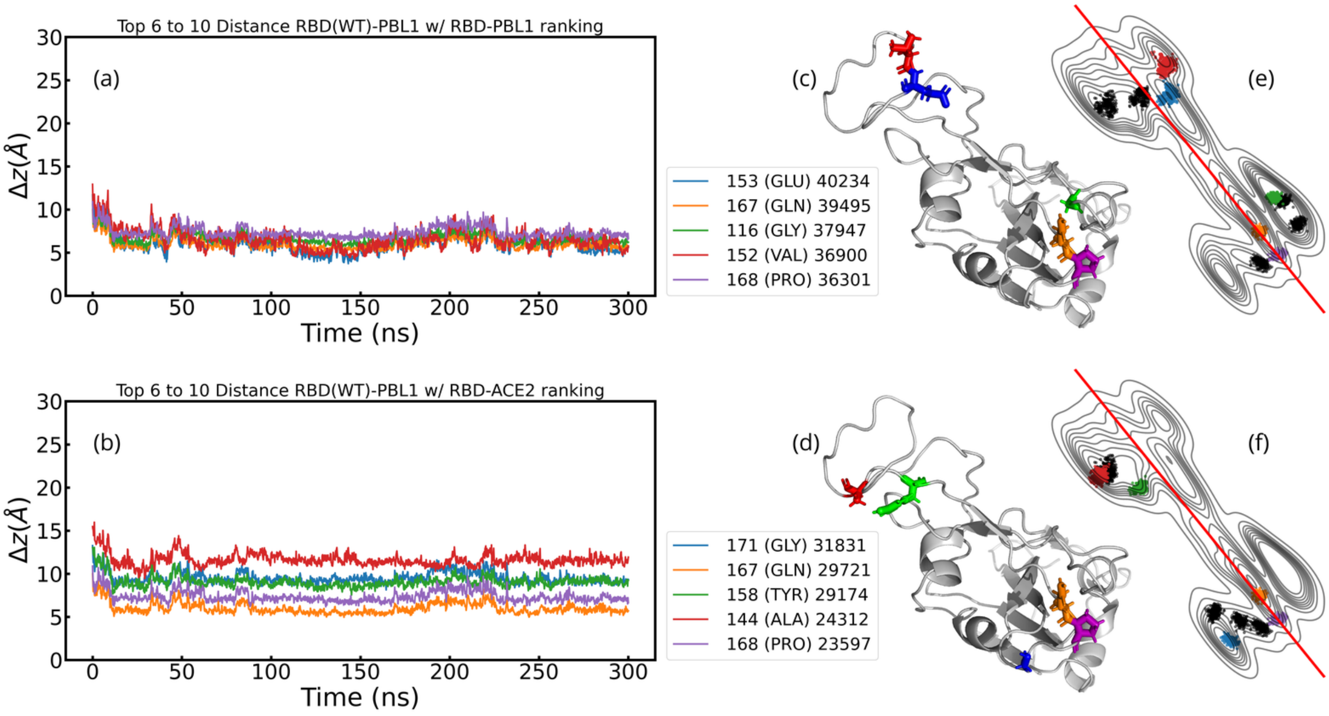

Figure S9: (a,b) Center of mass distance of the residues of WT-RBD to the hydrophilic substrate of the top 6 to 10 residues with most contacts with (a) the hydrophilic substrate and (b) the ACE2. Legends show the ResIDs, the residue names and the total contacts over the trajectory of each ranked residue (format: ResID-ResName TotalContacts).Visualization of residues loci are also shown in (c) and (d) with colors corresponding with the distance plots (a-b). Complementary to (d) and (e), we present KDE contour-line plots of top 10 residues with most contacts with (g) the hydrophobic substrate and (h) the ACE2. Note that in plots (g) and (h) the color code of the top 6 to 10 residues correspond to plots (d) and (e), the top 5 residues have adopted black color.

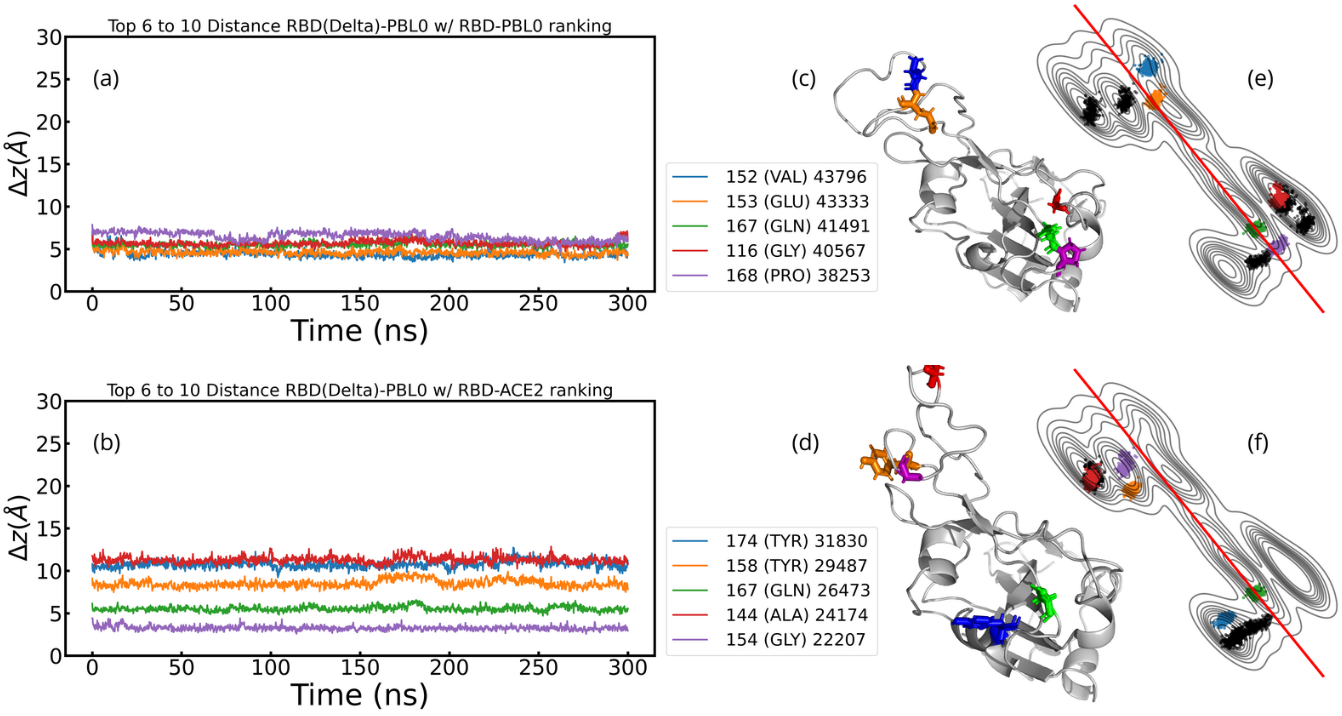

Figure S10: (a,b) Center of mass distance of the residues of Delta-RBD to the hydrophobic substrate of the top 6 to 10 residues with most contacts with (a) the hydrophobic substrate and (b) the ACE2. Legends show the ResIDs, the residue names and the total contacts over the trajectory of each ranked residue (format: ResID-ResName TotalContacts). Visualization of residues loci are also shown in (c) and (d) with colors corresponding with the distance plots (a-b).Complementary to (d) and (e), we present KDE contour-line plots of top 10 residues with most contacts with (g) the hydrophobic substrate and (h) the ACE2. Note that in plots (g) and (h) the color code of the top 6 to 10 residues correspond to plots (d) and (e), the top 5 residues have adopted black color.

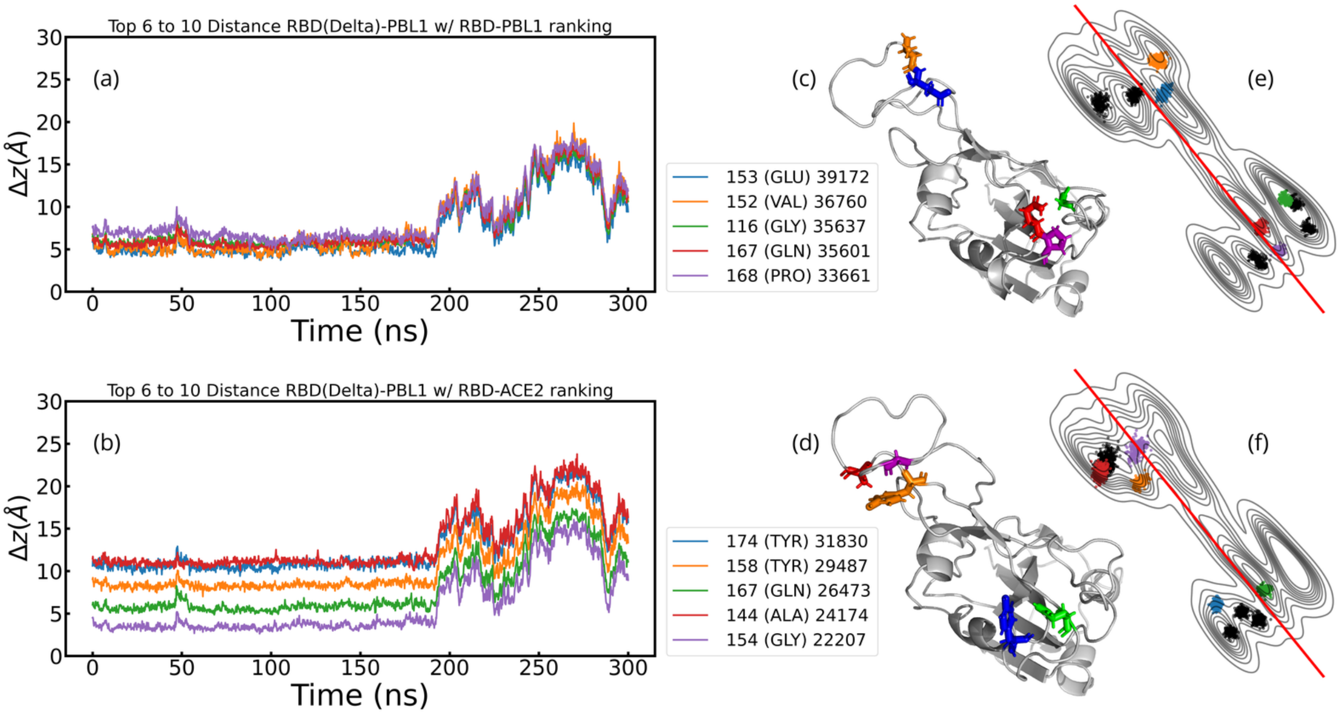

Figure S11: (a,b) Center of mass distance of the residues of Delta-RBD to the hydrophilic substrate of the top 6 to 10 residues with most contacts with (a) the hydrophilic substrate and (b) the ACE2. Legends show the ResIDs, the residue names and the total contacts over the trajectory of each ranked residue (format: ResID-ResName TotalContacts). Visualization of residues loci are also shown in (c) and (d) with colors corresponding with the distance plots (a-b).Complementary to (d) and (e), we present KDE contour-line plots of top 10 residues with most contacts with (g) the hydrophobic substrate and (h) the ACE2. Note that in plots (g) and (h) the color code of the top 6 to 10 residues correspond to plots (d) and (e), the top 5 residues have adopted black color.

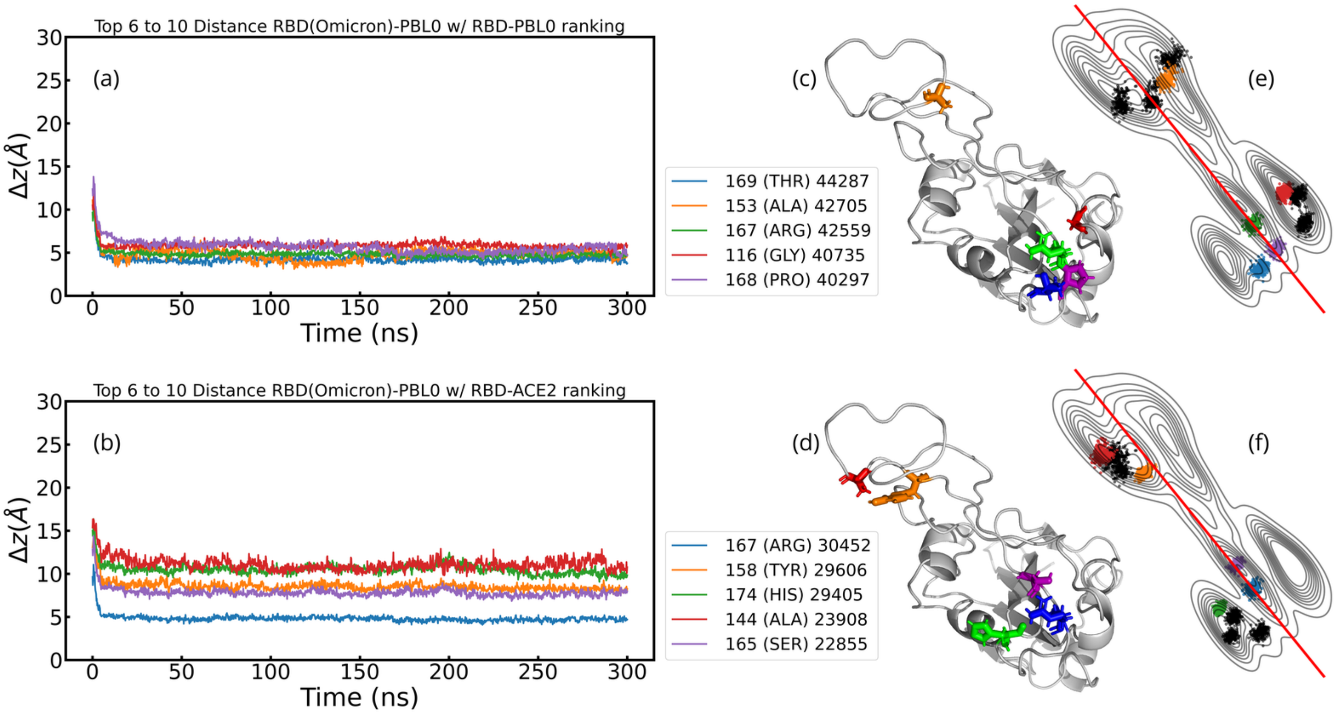

Figure S12: (a,b) Center of mass distance of the residues of Omicron-RBD to the hydrophobic substrate of the top 6 to 10 residues with most contacts with (a) the hydrophobic substrate and (b) the ACE2. Legends show the ResIDs, the residue names and the total contacts over the trajectory of each ranked residue (format: ResID-ResName TotalContacts). Visualization of residues loci are also shown in (c) and (d) with colors corresponding with the distance plots (a-b).Complementary to (d) and (e), we present KDE contour-line plots of top 10 residues with most contacts with (g) the hydrophobic substrate and (h) the ACE2. Note that in plots (g) and (h) the color code of the top 6 to 10 residues correspond to plots (d) and (e), the top 5 residues have adopted black color.

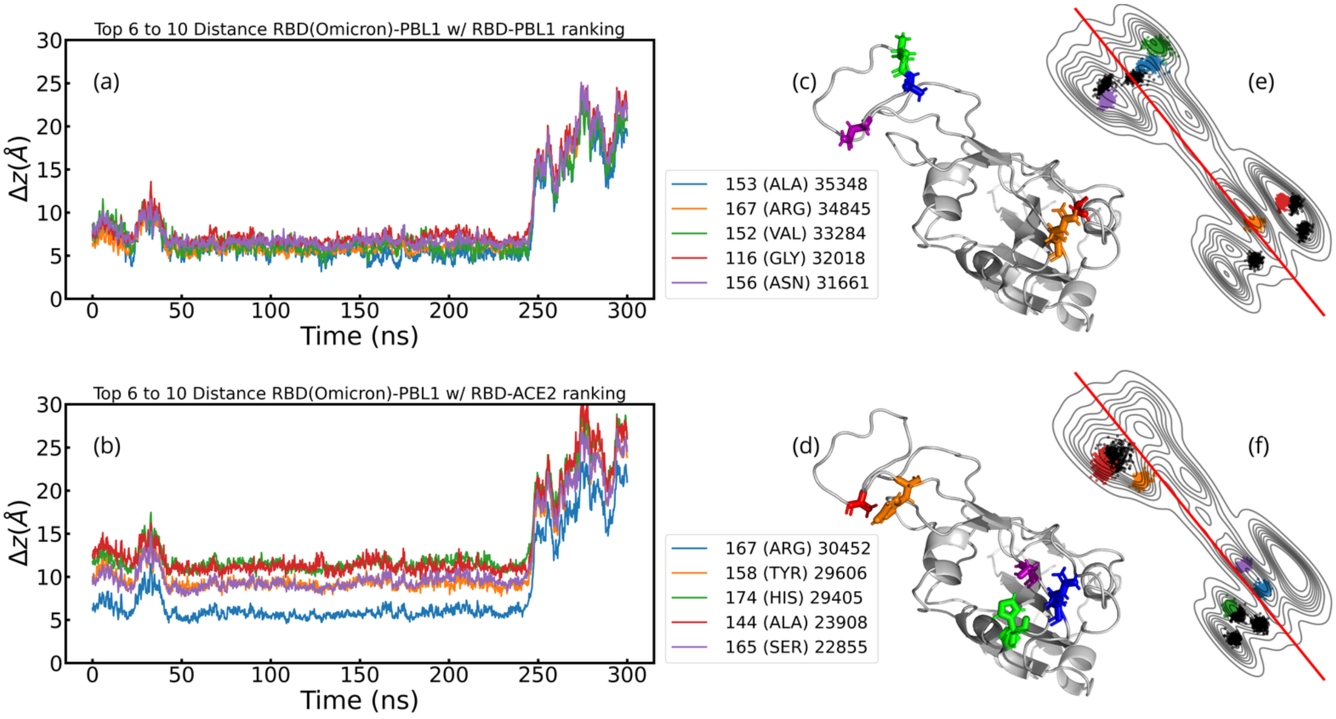

Figure S13: (a,b) Center of mass distance of the residues of Omicron-RBD to the hydrophilic substrate of the top 6 to 10 residues with most contacts with (a) the hydrophilic substrate and (b) the ACE2. Legends show the ResIDs, the residue names and the total contacts over the trajectory of each ranked residue (format: ResID-ResName TotalContacts). Visualization of residues loci are also shown in (c) and (d) with colors corresponding with the distance plots (a-b). Complementary to (d) and (e), we present KDE contour-line plots of top 10 residues with most contacts with (g) the hydrophobic substrate and (h) the ACE2. Note that in plots (g) and (h) the color code of the top 6 to 10 residues correspond to plots (d) and (e), the top 5 residues have adopted black color.

Table S10: Table contains the ResIDs and corresponding residue names of residues used in Group 2. Mutations are highlighted in fuchsia. **CN**: Charged Negative, **CP**: Charged Positive, **UP**: Uncharged Polar and **NP**: NonPolar

ResIDs	WT resnames	Delta resnames	Omicron resnames
111	ASP (CN)	ASP (CN)	ASP (CN)
112	SER (UP)	SER (UP)	SER (UP)
113	LYS (CP)	LYS (CP)	LYS (CP)
114	VAL (NP)	VAL (NP)	VAL (NP)
115	GLY (NP)	GLY (NP)	**SER (UP)**
116	GLY (NP)	GLY (NP)	GLY (NP)
118	TYR (UP)	TYR (UP)	TYR (UP)
117	ASN (UP)	ASN (UP)	ASN (UP)
161	LEU (NP)	LEU (NP)	LEU (NP)
162	GLN (UP)	GLN (UP)	**ARG (CP)**
163	SER (UP)	SER (UP)	SER (UP)
164	TYR (UP)	TYR (UP)	TYR (UP)
165	GLY (NP)	GLY (NP)	SER (UP)
166	PHE (NP)	PHE (NP)	PHE (NP)
167	GLN (UP)	GLN (UP)	**ARG (CP)**
168	PRO (NP)	PRO (NP)	PRO (NP)
169	THR (UP)	THR (UP)	THR (UP)
170	ASN (UP)	ASN (UP)	**TYR (UP)**
171	GLY (NP)	GLY (NP)	GLY (NP)
172	VAL (NP)	VAL (NP)	VAL (NP)
173	GLY (NP)	GLY (NP)	GLY (NP)
174	TYR (UP)	TYR (UP)	**HIS (CP)**

Table S11: Table contains the ResIDs and corresponding residue names and type of residues that define the Crook-handle for PBL0. Mutations are highlighted in fuchsia. Note that for omicron, this region is a hydrophobic pocket. Residue types are given by **CN**: Charged Negative, **CP**: Charged Positive, **UP**: Uncharged Polar and **NP**: NonPolar.

Polarity 0			
ResIDs	WT resnames	Delta resnames	Omicron resnames
150	ASN (UP)	ASN (UP)	ASN (UP)
151	GLY (NP)	GLY (NP)	GLY (NP)
152	VAL (NP)	VAL (NP)	VAL (NP)
153	GLU (CN)	GLU (CN)	**ALA (NP)**
154	GLY (NP)	GLY (NP)	GLY (NP)
155	PHE (NP)	PHE (NP)	PHE (NP)

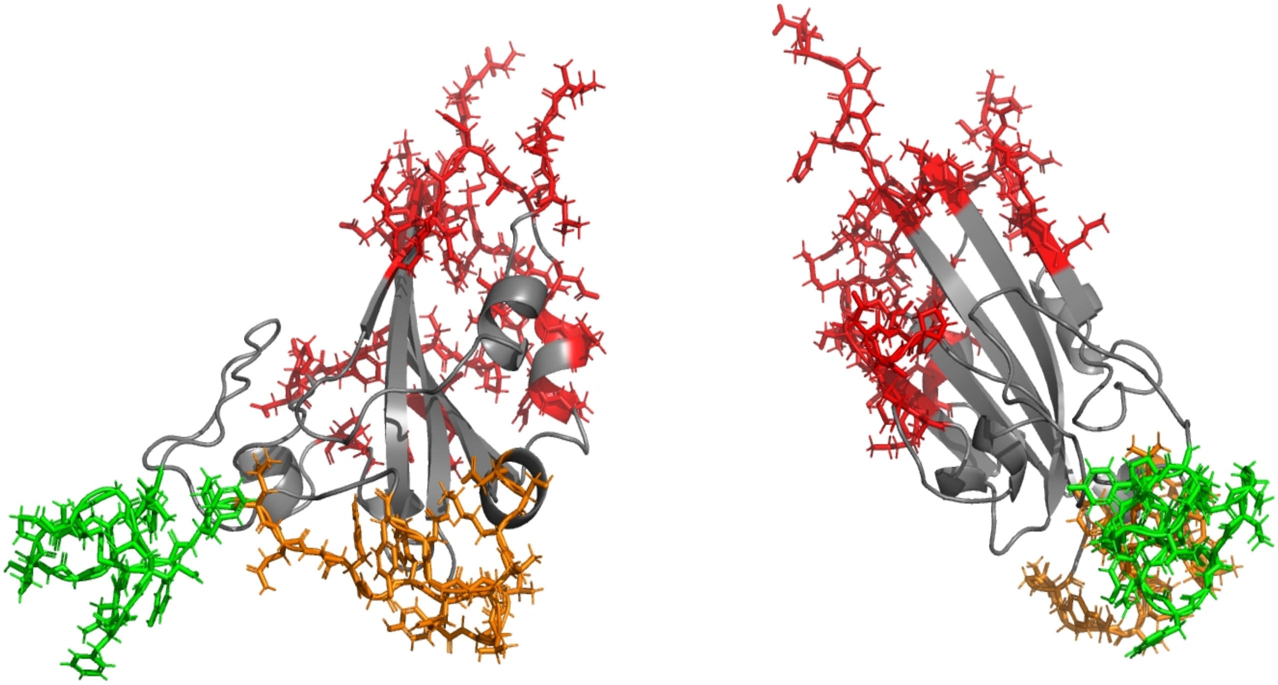

Figure S14: Front (left) and side view (right) of the RBD showing Group 1, Group 2 and restraint residues in green, orange and red, respectively.

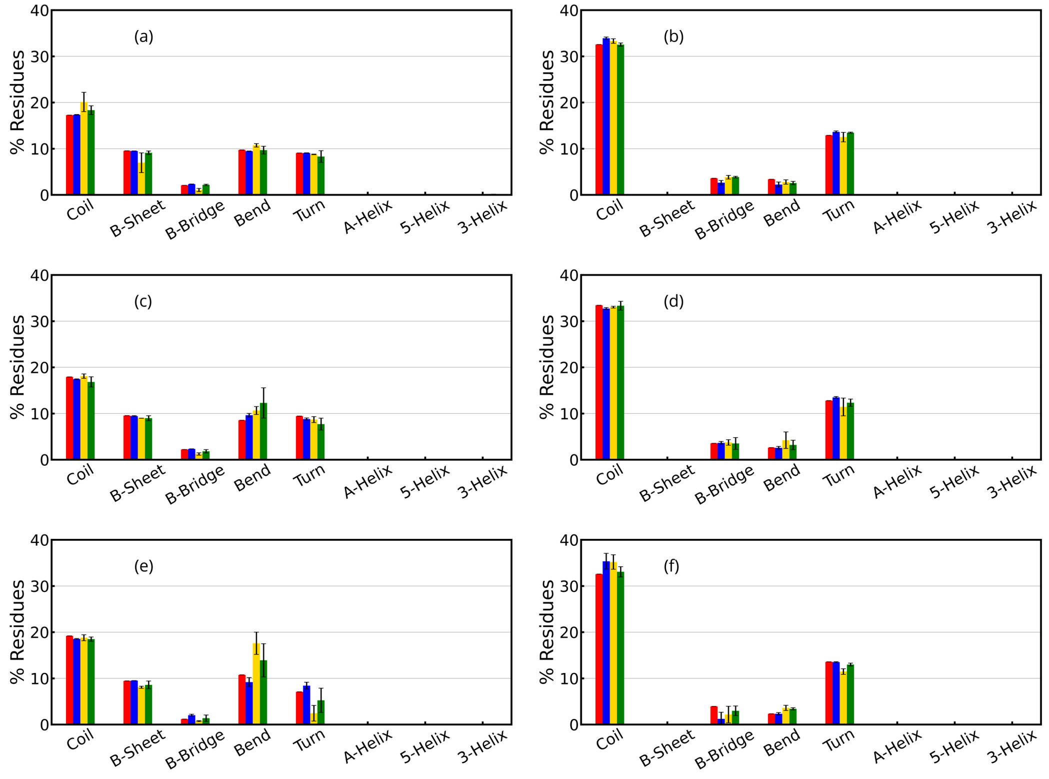

Figure S15: Secondary Structure percentage (SS %) of group 1 (left column) and group 2 (right column) averaged over the last 200 ns of the simulations (with the exception of the simulations of RBD alone, is the mean of all its 20 ns long trajectory) for (a,b) WT, (c,d) Delta, and (e,f) Omicron variants alone in water (red), with the ACE2 (blue), in presence of PBL0 (yellow), and PBL1 (green). Values are normalized over the number of residues in group 1 and 2.

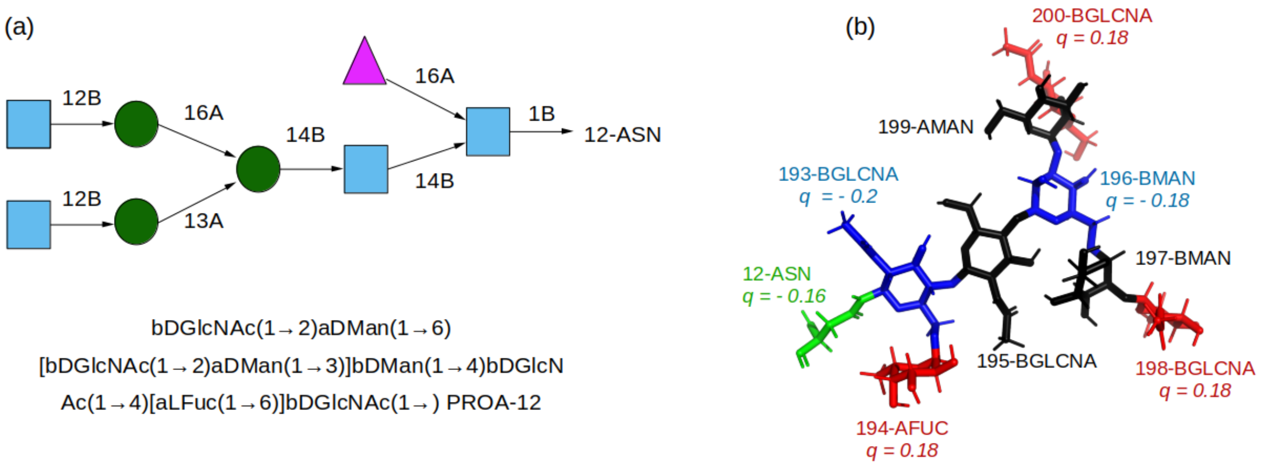

Figure S16: (a) Glycan Structure. (b) Glycan mapping and charge distribution. In (b), green is residue 12-ASN that binds the glycan to the protein, and the glycan is colored according the charge type. Red are positively charged, blue negatively charged and black are neutrally charged carbohydrates.

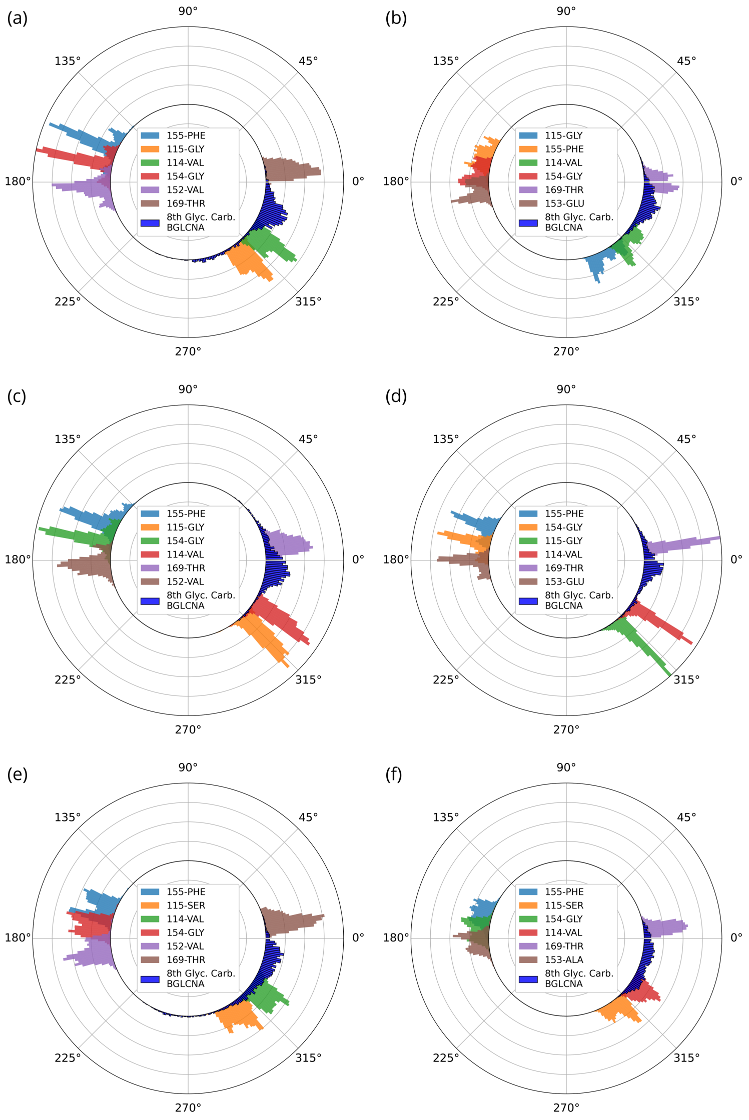

Figure S17: Angular Histogram of top contact residues of (a,b) WT, (c,d) Delta, and (e,f) Omicron. Center point is set at 20 Å distance from the mean position over time of each selected residue COM of the RBDs for each PBL (left column for hydrophobic surface and right for hydrophilic surfaces), and the glycan histogram from RBD-PBLs with Glycans simulations. Residues selected are Top 6 residues with most contacts to the PBLs, and the 8th residue of the glycan which is one of the further residues from the binding residue 12-ASN Schematic of the Glycan can be visualized in SI (Figure S16). Note that the first five colors are consistent to residue colors in Figure 11. Histogram is normalized over the maximum value for each surface, i.e. the maximum value of 169-THR in Delta-PBL0 and Omicron-PBL1.

Table S12: Table listing the ResIDs and corresponding residue names for each variant. Left and right legs are highlighted in green and orange rows, respectively. Mutations of delta and omicron referenced to wild-type of the RBD are shown with fuchsia highlighted cells. Fourth column indicates if residue is restraint in the model. It must be noted that ResIDs of the RBDs were initialized to one for practical reasons. Analogue values to PDB ID:6VSB requires adding up 331 units to the ResIDs,i.e. 1-ILE → 332-ILE (PDB) and 192-THR→ 523-THR.

ResIDs	WT	Delta	Omicron	Restraint
1	ILE	ILE	ILE	Yes
2	THR	THR	THR	Yes
3	ASN	ASN	ASN	Yes
4	LEU	LEU	LEU	Yes
5	CYS	CYS	CYS	No
6	PRO	PRO	PRO	No
7	PHE	PHE	PHE	No
8	GLY	GLY	**ASP**	No
9	GLU	GLU	GLU	No
10	VAL	VAL	VAL	No
11	PHE	PHE	PHE	No
12	ASN	ASN	ASN	No
13	ALA	ALA	ALA	No
14	THR	THR	THR	No
15	ARG	ARG	ARG	No
16	PHE	PHE	PHE	No
17	ALA	ALA	ALA	No
18	SER	SER	SER	No
19	VAL	VAL	VAL	No
20	TYR	TYR	TYR	No
21	ALA	ALA	ALA	No
22	TRP	TRP	TRP	No
23	ASN	ASN	ASN	No
24	ARG	ARG	ARG	No
25	LYS	LYS	LYS	Yes
26	ARG	ARG	ARG	Yes
27	ILE	ILE	ILE	Yes
28	SER	SER	SER	Yes
29	ASN	ASN	ASN	Yes
30	CYS	CYS	CYS	Yes
31	VAL	VAL	VAL	Yes
32	ALA	ALA	ALA	No
33	ASP	ASP	ASP	Yes
34	TYR	TYR	TYR	Yes
35	SER	SER	SER	Yes
36	VAL	VAL	VAL	Yes

ResIDs	WT	Delta	Omicron	Restraint
37	LEU	LEU	LEU	No
38	TYR	TYR	TYR	Yes
39	ASN	ASN	ASN	Yes
40	SER	SER	**LEU**	No
41	ALA	ALA	ALA	No
42	SER	SER	**PRO**	No
43	PHE	PHE	PHE	No
44	SER	SER	**PHE**	No
45	THR	THR	THR	No
46	PHE	PHE	PHE	No
47	LYS	LYS	LYS	Yes
48	CYS	CYS	CYS	Yes
49	TYR	TYR	TYR	Yes
50	GLY	GLY	GLY	Yes
51	VAL	VAL	VAL	Yes
52	SER	SER	SER	Yes
53	PRO	PRO	PRO	Yes
54	THR	THR	THR	Yes
55	LYS	LYS	LYS	Yes
56	LEU	LEU	LEU	Yes
57	ASN	ASN	ASN	Yes
58	ASP	ASP	ASP	Yes
59	LEU	LEU	LEU	Yes
60	CYS	CYS	CYS	Yes
61	PHE	PHE	PHE	Yes
62	THR	THR	THR	Yes
63	ASN	ASN	ASN	Yes
64	VAL	VAL	VAL	No
65	TYR	TYR	TYR	No
66	ALA	ALA	ALA	No
67	ASP	ASP	ASP	No
68	SER	SER	SER	No
69	PHE	PHE	PHE	No
70	VAL	VAL	VAL	No
71	ILE	ILE	ILE	No
72	ARG	ARG	ARG	No
73	GLY	GLY	GLY	No
74	ASP	ASP	ASP	No
75	GLU	GLU	GLU	No
76	VAL	VAL	VAL	No
77	ARG	ARG	ARG	No
78	GLN	GLN	GLN	No
79	ILE	ILE	ILE	No

ResIDs	WT	Delta	Omicron	Restraint
80	ALA	ALA	ALA	Yes
81	PRO	PRO	PRO	Yes
82	GLY	GLY	GLY	No
83	GLN	GLN	GLN	No
84	THR	THR	THR	No
85	GLY	GLY	GLY	No
86	LYS	LYS	ASN	No
87	ILE	ILE	ILE	No
88	ALA	ALA	ALA	No
89	ASP	ASP	ASP	No
90	TYR	TYR	TYR	No
91	ASN	ASN	ASN	No
92	TYR	TYR	TYR	No
93	LYS	LYS	LYS	No
94	LEU	LEU	LEU	No
95	PRO	PRO	PRO	Yes
96	ASP	ASP	ASP	Yes
97	ASP	ASP	ASP	Yes
98	PHE	PHE	PHE	Yes
99	THR	THR	THR	Yes
100	GLY	GLY	GLY	No
101	CYS	CYS	CYS	No
102	VAL	VAL	VAL	No
103	ILE	ILE	ILE	No
104	ALA	ALA	ALA	No
105	TRP	TRP	TRP	No
106	ASN	ASN	ASN	No
107	SER	SER	SER	No
108	ASN	ASN	ASN	No
109	ASN	ASN	LYS	No
110	LEU	LEU	LEU	No
111	ASP	ASP	ASP	No
112	SER	SER	SER	No
113	LYS	LYS	LYS	No
114	VAL	VAL	VAL	No
115	GLY	GLY	SER	No
116	GLY	GLY	GLY	No
117	ASN	ASN	ASN	No
118	TYR	TYR	TYR	No
119	ASN	ASN	ASN	No
120	TYR	TYR	TYR	No
121	LEU	ARG	LEU	No
122	TYR	TYR	TYR	No

ResIDs	WT	Delta	Omicron	Restraint
123	ARG	ARG	ARG	No
124	LEU	LEU	LEU	No
125	PHE	PHE	PHE	No
126	ARG	ARG	ARG	No
127	LYS	LYS	LYS	No
128	SER	SER	SER	No
129	ASN	ASN	ASN	No
130	LEU	LEU	LEU	No
131	LYS	LYS	LYS	No
132	PRO	PRO	PRO	No
133	PHE	PHE	PHE	No
134	GLU	GLU	GLU	No
135	ARG	ARG	ARG	No
136	ASP	ASP	ASP	No
137	ILE	ILE	ILE	No
138	SER	SER	SER	No
139	THR	THR	THR	No
140	GLU	GLU	GLU	No
141	ILE	ILE	ILE	No
142	TYR	TYR	TYR	No
143	GLN	GLN	GLN	No
144	ALA	ALA	ALA	No
145	GLY	GLY	GLY	No
146	SER	SER	**ASN**	No
147	THR	**LYS**	**LYS**	No
148	PRO	PRO	PRO	No
149	CYS	CYS	CYS	No
150	ASN	ASN	ASN	No
151	GLY	GLY	GLY	No
152	VAL	VAL	VAL	No
153	GLU	GLU	**ALA**	No
154	GLY	GLY	GLY	No
155	PHE	PHE	PHE	No
156	ASN	ASN	ASN	No
157	CYS	CYS	CYS	No
158	TYR	TYR	TYR	No
159	PHE	PHE	PHE	No
160	PRO	PRO	PRO	No
161	LEU	LEU	LEU	No
162	GLN	GLN	**ARG**	No
163	SER	SER	SER	No
164	TYR	TYR	TYR	No
165	GLY	GLY	**SER**	No

ResIDs	WT	Delta	Omicron	Restraint
166	PHE	PHE	PHE	No
167	GLN	GLN	**ARG**	No
168	PRO	PRO	PRO	No
169	THR	THR	THR	No
170	ASN	ASN	**TYR**	No
171	GLY	GLY	GLY	No
172	VAL	VAL	VAL	No
173	GLY	GLY	GLY	No
174	TYR	TYR	**HIS**	No
175	GLN	GLN	GLN	No
176	PRO	PRO	PRO	No
177	TYR	TYR	TYR	No
178	ARG	ARG	ARG	No
179	VAL	VAL	VAL	No
180	VAL	VAL	VAL	No
181	VAL	VAL	VAL	No
182	LEU	LEU	LEU	No
183	SER	SER	SER	No
184	PHE	PHE	PHE	No
185	GLU	GLU	GLU	Yes
186	LEU	LEU	LEU	Yes
187	LEU	LEU	LEU	Yes
188	HIS	HIS	HIS	Yes
189	ALA	ALA	ALA	Yes
190	PRO	PRO	PRO	Yes
191	ALA	ALA	ALA	Yes
192	THR	THR	THR	Yes
